# Loss of SHP1 in Spinal Astrocytes Triggers T‐Lymphocyte Infiltration and Nociceptive Hypersensitivity

**DOI:** 10.1002/advs.76932

**Published:** 2026-08-03

**Authors:** Lan‐Xing Yi, Lin Yang, Kang‐Li Wang, Hui‐Zhu Liu, Rui‐Ying Chen, Min Su, Xiao Xiao, Yu‐Qiu Zhang

**Affiliations:** ^1^ Department of Translational Neuroscience Jing'an District Centre Hospital of Shanghai State Key Laboratory of Brain Function and Disorders and MOE Frontiers Center For Brain Science Institutes of Brain Science Fudan University Shanghai China; ^2^ Department of Endocrinology Huadong Hospital; Key Laboratory of Computational Neuroscience and Brain‐Inspired Intelligence Ministry of Education; Behavioral and Cognitive Neuroscience Center Institute of Science and Technology for Brain‐Inspired Intelligence MOE Frontiers Center for Brain Science Fudan University Shanghai China; ^3^ Department of Pathology and Pathophysiology Shantou University Medical College Shantou China

**Keywords:** allodynia, astrocyte, blood‐brain barrier, CXCL10/CXCR3, SHP1, T cell infiltration

## Abstract

Chronic pain is sustained by complex neuroimmune interplay within the spinal cord, yet how astrocytes constrain immune amplification remains poorly understood. Here we identified Src‐homology 2 domain‐containing protein tyrosine phosphatase‐1 (SHP1; encoded by *Ptpn6*) as a key suppressor of spinal neuroinflammation and nociceptive hypersensitivity. Astrocyte‐specific SHP1 deletion in the spinal dorsal horn induced profound astrocytic morphological changes, increased C‐X‐C motif chemokine ligand 10 (CXCL10) expression, disrupted blood‐brain barrier (BBB) integrity, and facilitated the infiltration of activated T lymphocytes into the spinal parenchyma. These infiltrating T cells promoted microglial activation, enhanced excitatory synaptic transmission, increased the excitability of somatostatin (SOM)‐positive neurons, and ultimately triggered nociceptive hypersensitivity. Mechanistically, T cell recruitment was governed by an SHP1‐dependent regulatory circuit, in which SHP1‐mediated Signal transducer and activator of transcription 1 (STAT1) dephosphorylation restrained *Cxcl10* transcription. Blocking the STAT1–CXCL10–CXCR3 signaling axis alleviated T cell infiltration and nociceptive hypersensitivity. Notably, we found that during the development of neuropathic pain, spinal SHP1 expression was significantly downregulated, accompanied by upregulation of CXCL10. Overexpression of SHP1 in astrocytes markedly attenuated astrocyte activation and alleviated mechanical allodynia induced by spared nerve injury (SNI). Collectively, our findings revealed an astrocyte‐defined immune checkpoint that restrains neuroinflammatory escalation and pain persistence, emphasizing SHP1 as a promising therapeutic target for chronic pain.

## Graphical abstract

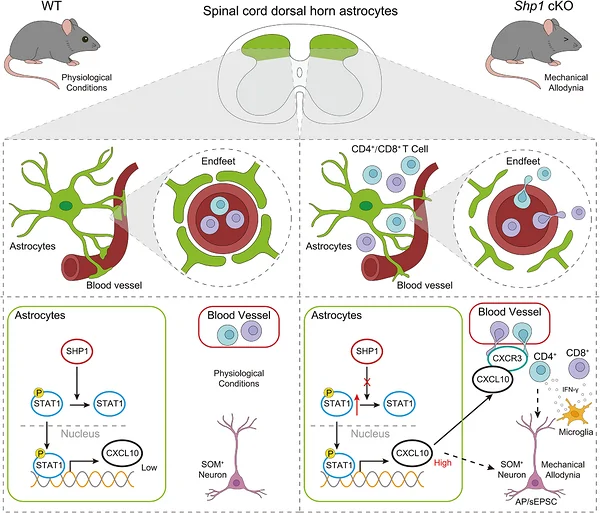

## 1 Introduction

Recent studies emphasize the pivotal role of spinal astrocytes in modulating nociceptive processing. Optogenetic activation of spinal astrocytes directly induces pain hypersensitivity [1, 2]. In chronic pain conditions, activated spinal astrocytes release various proinflammatory cytokines and chemokines, contributing to central sensitization [3, 4]. These findings identify spinal astrocytes as key regulators of pain hypersensitivity. Over the past two decades, the concept of a dynamic glia‐neuron‐immune cell network has gained prominence, emphasizing the influence of immune responses on pain pathways [5]. Peripheral immune cells, particularly T lymphocytes, infiltrate the central nervous system (CNS) following injury and promote the development and persistence of neuropathic pain [6]. However, the mechanisms driving T lymphocyte infiltration and the potential involvement of astrocytes in this process remain uncertain.

Src homology region 2 domain‐containing phosphatase‐1 (SHP1), encoded by the *Ptpn6* gene, is a conserved protein tyrosine phosphatase expressed in humans and mice [7]. Multiple studies have established SHP1 as a critical negative regulator of multiple immune signaling pathways in peripheral immune cells [8]. SHP1 constitutively associates with the T‐cell receptor (TCR) and B‐cell receptor (BCR), modulating activation thresholds through the dephosphorylation of the TCR‐ζ chain, and downstream signaling proteins including Lck, ZAP70, Vav, and PI3K [9, 10]. Inhibition of SHP1 lowers the TCR activation threshold, expanding T‐lymphocyte repertoire [11], whereas *Ptpn6* overexpression reduces the splenic T‐cell proportion, and suppresses proliferation [12]. Within the CNS, astrocytes exhibit immune‐like properties, including secretion of diverse chemokines and cytokines, regulation of blood‐brain barrier (BBB) integrity, and structural contribution to the BBB [13]. SHP1 deficiency leads to a pronounced increase in reactive astrocytes in the brain, characterized by upregulated glial fibrillary acidic protein (GFAP) expression [14]. However, whether SHP1 directly regulates astrocytic immune functions within the CNS remains unclear. Notably, SHP1 has also been implicated in pain modulation. For instance, SHP1 dephosphorylates transient receptor potential vanilloid 1 (TRPV1) in dorsal root ganglion (DRG) neurons, alleviating complete Freund's adjuvant (CFA)‐induced inflammatory pain [15]. Studies from our group and collaborators have identified SHP1 as a key downstream mediator of programmed death‐ligand 1 analgesia, acting through sodium channels, TREK2 K^+^ channels, and TRPV1 channels in DRG neurons under acute and chronic pain conditions [16, 17]. Furthermore, SHP1 mediates the analgesia induced by voltage‐gated proton channel (Hv1) inhibition in DRG neurons [18]. To date, investigations into SHP1's role in nociceptive transmission have focused primarily on peripheral sensory neurons, whereas its function within the CNS—particularly in non‐neuronal cells, such as astrocytes—remains uncertain. Considering SHP1's well‐established immunoregulatory functions and the recognized involvement of astrocytes in pain modulation, we hypothesize that SHP1 contributes to spinal nociception through astrocyte‐mediated immunomodulatory mechanisms.

We combined genetic manipulation, single‐nucleus RNA sequencing, cleavage under targets and tagmentation (CUT&Tag), and functional analyses to investigate the role of SHP1 in spinal astrocytes. We detected SHP1 expression in spinal astrocytes, and selective ablation of SHP1 in these cells increased spinal infiltration of T lymphocytes, enhancing the excitability of spinal dorsal horn (SDH) somatostatin‐positive (SOM^+^) neurons and inducing nociceptive hypersensitivity. Furthermore, we investigated the impact of astrocyte‐specific SHP1 knockout on the immune‐like properties of astrocytes.

## 2 Results

### 2.1 SHP1 Deficiency in Spinal Astrocytes Induces Mechanical Allodynia in Mice

In the superficial laminae (laminae I‒III) of SDH, SHP1 immunofluorescence was observed predominantly in GFAP^+^ astrocytes, partially in Nissl^+^ neurons, and minimally in Iba‐1^+^ microglia (Figure 1A and Figure S1A‒C). Co‐expression of SHP1 and GFAP was confirmed in human SDH, supporting clinical relevance (Figure S1D). To investigate the role of astrocytic SHP1 in spinal nociception, Cre recombinase‐expressing adeno‐associated virus (AAV) driven by the gfaABC1D promoter (pAAV‐gfaABC1D‐EGFP‐P2A‐Cre) was injected into L3‐L5 SDH of *Ptpn6* ^flox/flox^ mice to genetically delete *Ptpn6* in lumbar SDH astrocytes, referred to as *Ptpn6* conditional knockout (cKO) (Figure 1B and Figure S1E,F). *Ptpn6* cKO mice exhibited significantly reduced paw withdrawal thresholds (PWTs) and increased paw withdrawal frequencies (PWFs) in response to von Frey filament stimulation in both male and female mice (Figure 1C, D and Figure S1G,H). In contrast, neuron‐specific SHP1 deletion, achieved by intra‐dorsal horn of njecting the human synapsin 1 (hSyn) promoter‐driven Cre virus (AAV‐hSyn‐Cre‐EGFP) into the SDH of *Ptpn6* ^flox/flox^ mice, did not alter the paw withdrawal threshold and response frequency to *von* Frey stimulation (Figure S1I,J). Furthermore, pharmacological inhibition of SHP1 via intrathecal administration of PTP inhibitor III (PTPi, a SHP1 inhibitor) also produced robust mechanical allodynia in both hindpaws (Figure S1K). These findings highlight the critical involvement of spinal astrocytic SHP1 in spinal nociceptive modulation.

**FIGURE 1:**
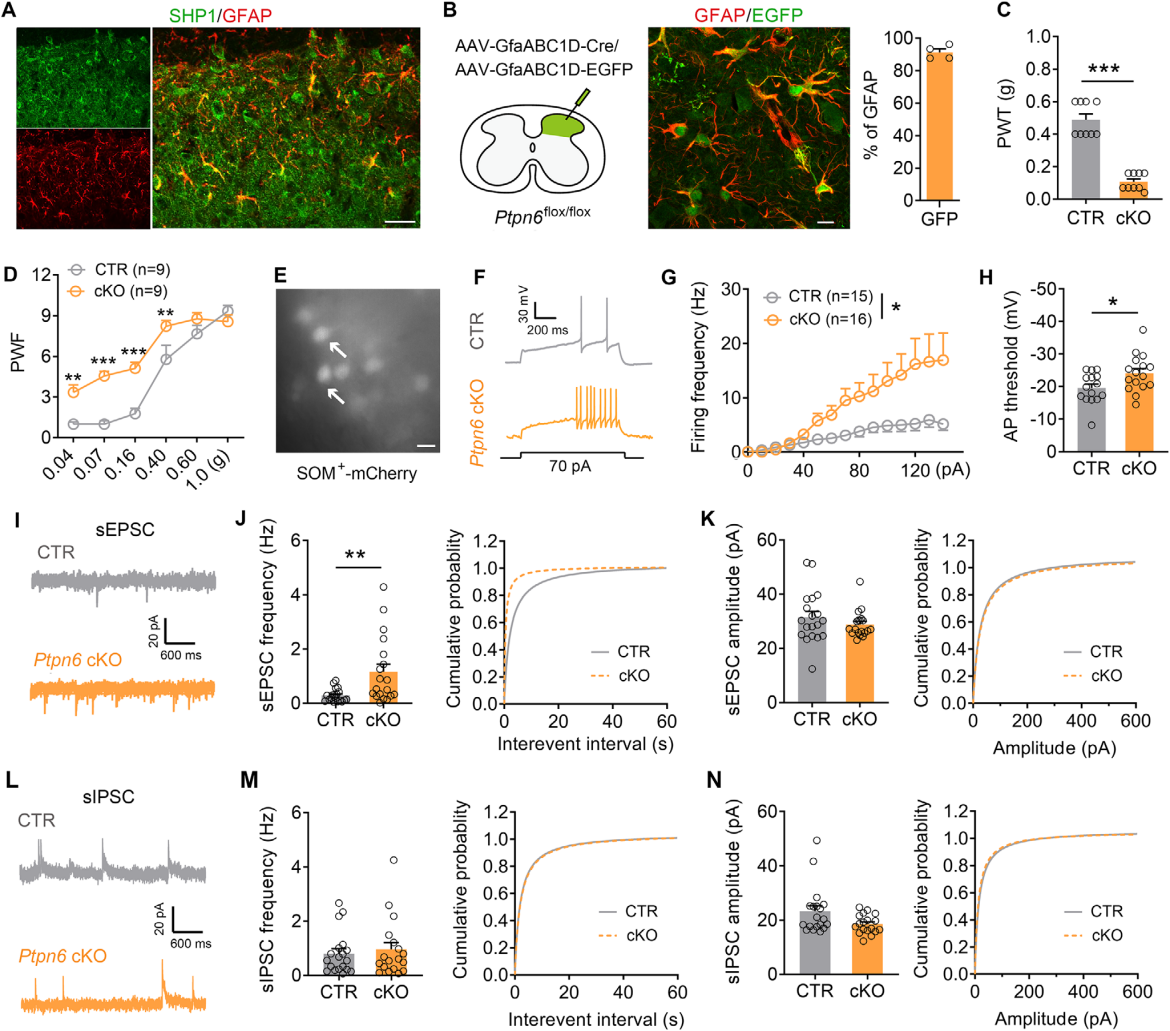
Astrocyte‐specific SHP1 deficiency drives mechanical allodynia by heightening the excitability and excitatory synaptic transmission of SOM+ neurons in the spinal dorsal horn. (A) Representative immunofluorescence image showing co‐localization of SHP1 and GFAP in superficial laminae of the SDH. Scale bar, 20 µm. (B) Schematic of the strategy for astrocyte‐specific SHP1 deletion via viral infection of GfaABC1D‐Cre‐EGFP on the SDH in *Ptpn6* ^flox/flox^ mice, and virus‐specific validation. Scale bar, 20 µm. n = 4. (C and D) Paw withdrawal threshold (PWT, C) and paw withdrawal frequency (PWF, D) in responses to *von* Frey stimulation of mice treated with astrocyte‐specific SHP1 deletion (cKO) and control (CTR). n = 9. (E) Representative mouse spinal cord slice showing recorded SOM^+^ neurons in lamina IIo of spinal dorsal horn (labeled by mCherry). Scale bar, 10 µm. (F) Representative traces of action potentials (APs) in spinal SOM^+^ neurons in different treatment groups. (G and H) Quantification of AP firing frequencies (G) and AP threshold (H) in spinal SOM^+^ neurons from control (n = 15, cells) and cKO (n = 16, cells) from four mice per group. (I) Representative traces of sEPSCs in spinal SOM^+^ neurons from different treatment groups. (J and K) Quantification of frequency (J) and amplitude (K) of sEPSC and corresponding cumulative distribution in spinal SOM^+^ neurons. n = 19 cells from four mice per group. (L) Representative traces of sIPSCs in spinal SOM^+^ neurons from different treatment groups. (M and N) Quantification of frequency (M) and amplitude (N) of sIPSC and corresponding cumulative distribution in SOM^+^ neurons. n = 18–19 cells from four mice per group. All data are presented as mean ± SEM. ^*^ *p* < 0.05, ^**^ *p* < 0.01, ^***^ *p* < 0.001.

Somatostatin‐positive (SOM^+^) neurons in the SDH have been implicated in mediating mechanical hypersensitivity [19, 20]. These neurons exhibit remarkable changes in neuronal excitability and synaptic plasticity under neuropathic pain conditions [21]. To determine whether the astrocytic *Ptpn6* deletion affects SOM^+^ neuronal activity, we generated Som^flpo^::*Ptpn6* ^flox/flox^ mice anco‐injected an astrocyte‐specific Cre‐expressing virus (pAAV‐gfaABC1D‐EGFP‐P2A‐Cre) together with AAV‐fDIO‐mCherry into the SDH to selectively delete *Ptpn6* in astrocytes while selectively labeling SDH SOM^+^ neurons (Figure 1E). Whole‐cell patch‐clamp recordings revealed that input‒output curves of action potentials (APs) were significantly left‐shifted, accompanied by a reduced AP firing threshold in *Ptpn6* cKO mice (Figure 1F‒H). No significant differences were observed in resting membrane potential (RMP), membrane resistance (Rm), membrane capacitance (Cm), membrane time constant (Tau), AP rheobase, AP amplitude and half width, or afterhyperpolarization (AHP) amplitude and latency between groups (Figure S2A‒I). We next recorded spontaneous excitatory postsynaptic currents and spontaneous inhibitory postsynaptic currents (sEPSCs and sIPSCs) in outer lamina II (II_o_) SOM^+^ neurons of the SDH. Astrocytic SHP1 deficiency induced a significant increase in frequency of sEPSCs, indicating enhanced excitatory synaptic transmission in lamina II_o_ SOM^+^ neurons (Figure 1I‒K). In contrast, sIPSCs did not differ significantly between control and *Ptpn6* cKO mice (Figure 1L–N). To verify further the contribution of SOM^+^ neurons in astrocytic SHP1 deficiency‐induced allodynia, we chemogenetically inhibited SOM^+^ neurons in *Ptpn6* cKO mice (Figure S2J,K). Inhibition of SDH SOM^+^ neurons significantly attenuated the allodynia induced by astrocytic SHP1 deficiency (Figure S2L), suggesting that *Ptpn6* cKO‐induced allodynia is mediated by increased excitability and enhanced excitatory synaptic transmission of SOM^+^ neurons.

### 2.2 Loss of SHP1 Promotes Astrocytic Process Complexity and Leads to BBB Leakage

Considering the established role of SHP1 in regulating glial activation in the brain and the involvement of spinal astrocytes in nociceptive processing, we next examined astrocytic reactions in the SDH of *Ptpn6* cKO mice. Morphologically, astrocytes lacking *Ptpn6* exhibited increased complexity, characterized by enhanced GFAP fluorescence intensity, a greater number of intersections in Sholl analysis, and prolonged and more abundant processes (Figure 2A–F). Consistently, Western blot analysis showed an upregulated GFAP level in the *Ptpn6* cKO SDH (Figure 2G). These findings indicate that *Ptpn6* deficiency in astrocytes promotes their transition to a reactive phenotype.

**FIGURE 2:**
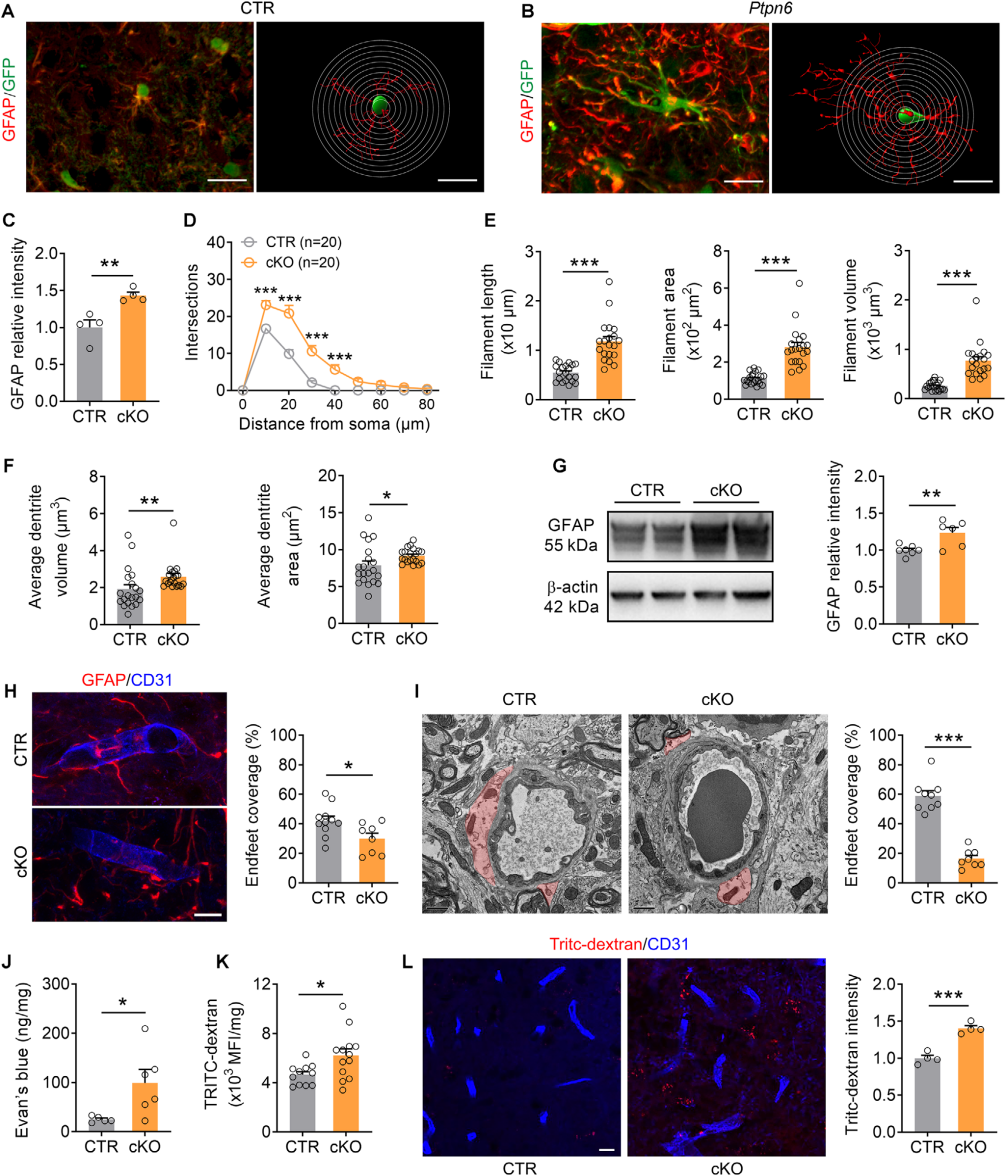
Astrocyte‐specific SHP1 deletion triggers astrocyte reactivity and disrupts BBB integrity. (A, B) Representative immunohistochemistry images showing astrocyte morphology of control (A) and *Ptpn6* cKO (B) mice. Scale bars, 20 µm. (C) Quantification of GFAP fluorescence intensity in the dorsal horn. n = 4. (D) Sholl analysis of spinal astrocyte arborization in control and cKO groups. n = 20 cells from four mice per group. (E, F) Morphometric analysis of spinal astrocytes for filament length, area, volume, and average dendritic volume and area (F). n = 20 cells from four mice per group. (G) Western blot analysis of GFAP protein expression in the spinal dorsal horn. n = 6–7. (H, I) Immunohistochemistry (H) and transmission electron microscopy (I) analysis of the coverage of blood vessels by astrocytic endfeet. Scale bars, 10 µm (H); 2 µm (I). n = 8–11 (vessels) from three mice per group. (J, K) Quantification of Evans blue (J) and dextran (K) leakage into the spinal dorsal horn. n = 11–24. (L) Immunohistochemistry staining showing dextran leakage in the spinal dorsal horn parenchyma in cKO mice. Scale bar, 10 µm. n = 4. All data are presented as mean ± SEM. ^*^ *p* < 0.05, ^**^ *p *< 0.01, ^***^ *p* < 0.001.

Cell morphology is largely governed by cytoskeletal proteins such as F‐actin. Previous reports have shown that F‐actin is a direct SHP1 substrate in B cells, where *Ptpn6‐*mediated dephosphorylation is essential for actin depolymerization [22]. We confirmed the interaction between SHP1 and F‐actin by co‐immunoprecipitation (Co‐IP) assays in primary cultured spinal astroglial cells (Figure S3A) and the mouse glioma cell line (G422) (Figure S3B). Phalloidin staining revealed increased F‐actin accumulation following SHP1 inhibition with the PTPi treatment in primary cultured spinal astroglial cells (Figure S3C). Consistently, transfection with *Ptpn6‐*shRNA and F‐actin‐EGFP co‐expression vectors into the G422 cells resulted in enhanced F‐actin polymerization in the *Ptpn6* knockdown cells (Figure S3D,E). Moreover, astrocytic *Ptpn6* cKO upregulated phosphorylated VAV guanine nucleotide exchange factor 3 (pVAV3), a VAV isoform predominantly expressed in SDH astrocytes (Figure S3F). These findings support the notion that SHP1 modulates cytoskeletal reorganization in astrocytes, at least in part, through the regulation of F‐actin and VAV3 phosphorylation [23].

Astrocytic endfeet closely appose the abluminal surface of microvascular endothelial cells, a structural arrangement essential for maintaining the integrity and function of the BBB. Morphological alterations in astrocytes may disrupt this intimate association, increasing BBB permeability. To determine whether *Ptpn6* deficiency affects astrocyte–vessel interactions, we quantified astrocytic endfeet coverage on microvessels in the SDH of *Ptpn6* cKO and control mice. Immunofluorescence and transmission electron microscopy (TEM) assessments revealed a pronounced reduction in endfeet coverage in *Ptpn6* cKO mice (Figure 2H,I). BBB disruption may be associated with abnormal reduction or aberrant upregulation of endothelial tight junction proteins. Thus, we detected the expression of Claudin‐5, Occludin, and ZO‐1 in the SDH. Western blotting revealed markedly aberrant expression of the three tight junction proteins following SHP1 cKO (Figure S3G–J). We next assessed macromolecular leakage across the BBB, Evans blue and tetramethylrhodamine isothiocyanate (TRITC)‐dextran were intravenously administered, and their extravasation within the SDH was evaluated. Quantitative analysis demonstrated significantly increased Evans blue and TRITC‐dextran leakage in *Ptpn6* cKO mice compared to controls (Figure 2J–L). These findings indicate that astrocytic SHP1 deficiency compromises BBB integrity in the SDH, likely through increased astrocytic morphological complexity and impaired endfeet‐vessel interactions.

### 2.3 Single‐Nucleus RNA Sequencing (snRNA‐seq) Reveals Spinal T Lymphocyte Infiltration in *Ptpn6* cKO Mice

The BBB functions as a unique “immune barrier”, restricting the entry of peripheral immune cells and numerous large molecules, such as antibodies, complement, and cytokines, that passg from the circulation into the spinal cord and brain parenchyma. As shown above, *Ptpn6* cKO mice exhibited increased BBB permeability and nociceptive sensitization, suggesting the presence of a proinflammatory CNS environment, involving T cell infiltration into the SDH. Therefore, we performed snRNA‐Seq analysis on SDH tissue from control and *Ptpn6* cKO mice (Figure 3A and Figure S4A). t‐distributed stochastic neighbor embedding (t‐SNE) analysis identified nine distinct cell clusters (Figure 3B), which were annotated according to established marker genes: *Gfap*, *Aldh1l1* and *Sox9* for astrocytes; *Rbfox3* for neurons; *Sox10* for oligodendrocytes; *Cx3cr1* for microglia; and *Cd3d* for T lymphocytes, among others (Figure S4B).

**FIGURE 3:**
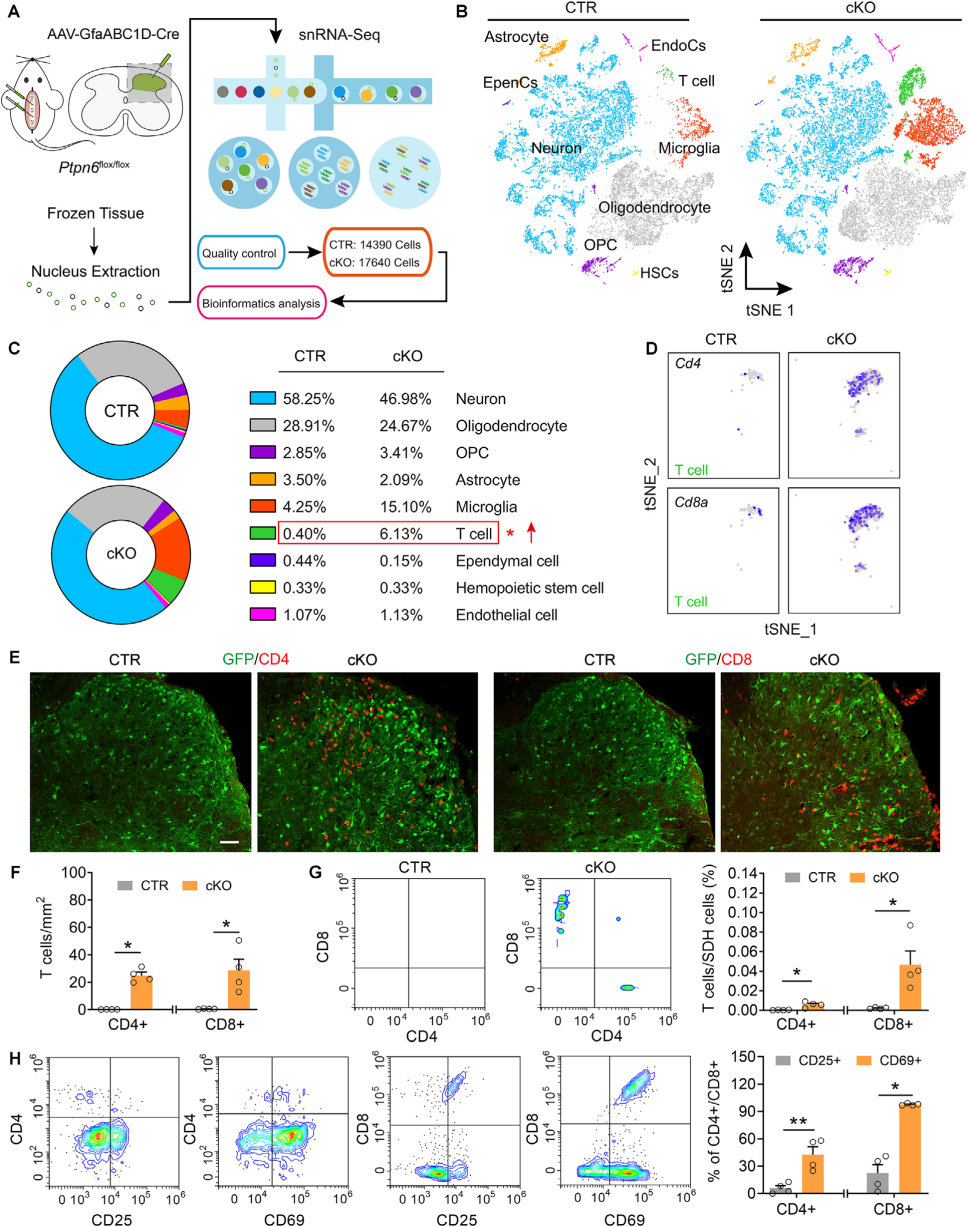
Astrocyte‐specific SHP1 deficiency leads to T cell infiltration into the spinal dorsal horn parenchyma. (A) Schematic diagram illustrating the workflow of single‐nucleus RNA sequencing analysis. (B) t‐SNE visualization showing cell clustering in the spinal dorsal horn of control and cKO groups. (C) Quantification of major cell populations in the spinal dorsal horn of control and cKO groups. (D) Proportions of CD4^+^ and CD8^+^ T lymphocytes based on scRNA‐seq data. (E) Representative immunofluorescence images showing CD4^+^ and CD8^+^ T cell infiltration into the spinal dorsal horn in cKO mice. Scale bar, 50 µm. (F) Quantification of CD4^+^ and CD8^+^ T cell numbers in the spinal dorsal horn. n = 4. (G) Representative flow cytometry plots and quantification of CD4^+^ and CD8^+^ T cell populations. n = 4. (H) Quantification of the proportions of CD25^+^ and CD69^+^ cells among CD4^+^ and CD8^+^ T lymphocytes. n = 4. All data are presented as mean ± SEM. ^*^ *p* < 0.05, ^**^ *p* < 0.01.

*Ptpn6* cKO mice exhibited a pronounced increase in T lymphocyte clusters within the SDH (Figure 3C). These infiltrating lymphocytes were predominantly CD4^+^ and CD8^+^ T cells (Figure 3D). Consistent with the snRNA‐seq findings, immunohistochemical staining revealed marked infiltration of CD4^+^ and CD8^+^ T cells in the spinal cord of *Ptpn6* cKO mice (Figure 3E,F). Flow cytometric analysis further validated these observations (Figure 3G and Figure S4C). Notably, the infiltrating CD4^+^ and CD8^+^ T cells were largely CD69^+^, an activation marker, indicating that most T cells were in an activated state (Figure 3H). To explore astrocytic transcriptional changes further, we analyzed differentially expressed genes (DEGs) in SDH astrocytes between *Ptpn6* cKO and control mice. Gene ontology analysis of biological processes revealed significant enrichment in immune‐related pathways, including “regulation of innate immune response”, “regulation of immune effector process”, “positive regulation of adaptive immune response”, and “antigen processing and presentation” (Figure S5A). In total, 220 DEGs were identified, of which 201 were upregulated, and 19 were downregulated following astrocytic *Ptpn6* deletion (Figure S5B). These findings emphasize the pivotal role of SHP1 in modulating astrocytic immune functions within the spinal cord.

### 2.4 Astrocyte‐specific *Ptpn6* Deficiency‐Induced Spinal T Lymphocyte Infiltration Drives Mechanical Allodynia

To determine whether the SDH‐infiltrating CD4^+^ T and CD8^+^ T lymphocytes mediate *Ptpn6* cKO‐induced mechanical allodynia, T cells were depleted by intraperitoneal injection of T cell neutralizing antibodies every 3 days (q3d) for a total of eight doses. Following anti‐T treatment, the numbers of infiltrating CD4^+^ and CD8^+^ T cells in the SDH of *Ptpn6* cKO mice were significantly reduced (Figure 4A–C). Behaviorally, the CD4 and CD8 neutralizing antibody cocktail (anti‐T, 100 µg of each) or CD4 neutralizing antibody alone (100 µg) both potently ameliorated *Ptpn6* cKO‐induced mechanical allodynia, whereas CD8 neutralizing antibody alone had no effect (Figure 4D). This finding indicates that CD4^+^ T cells are necessary for the mechanical allodynia resulting from *Ptpn6* cKO. Electrophysiologically, anti‐T effectively reversed *Ptpn6* cKO‐induced neuronal hyperexcitability, and increased frequency of sEPSCs in spinal SOM^+^ neurons (Figure 4E–J). Moreover, T cell depletion also partially suppressed astrocyte *Ptpn6* deficiency‐induced microglial activation (Figure 5A‒C). Previous studies have demonstrated that CD4^+^ T cells infiltrating the SDH secrete interferon (IFN)‐γ to activate microglia, contributing to neuropathic pain [24]. Consistent with this, we further confirmed IFN‐γ expression in infiltrating T lymphocytes within the SDH of *Ptpn6* cKO mice (Figure 5D), and administration of an IFN‐γ neutralizing antibody (3 µg) significantly alleviated mechanical allodynia and reduced Iba‐1 expression (Figure 5E‒G). These findings suggest that IFN‐γ mediates the crosstalk between infiltrating T lymphocytes and microglia in the SDH, contributing to astrocytic *Ptpn6* deficiency‐induced nociceptive hypersensitivity.

**FIGURE 4:**
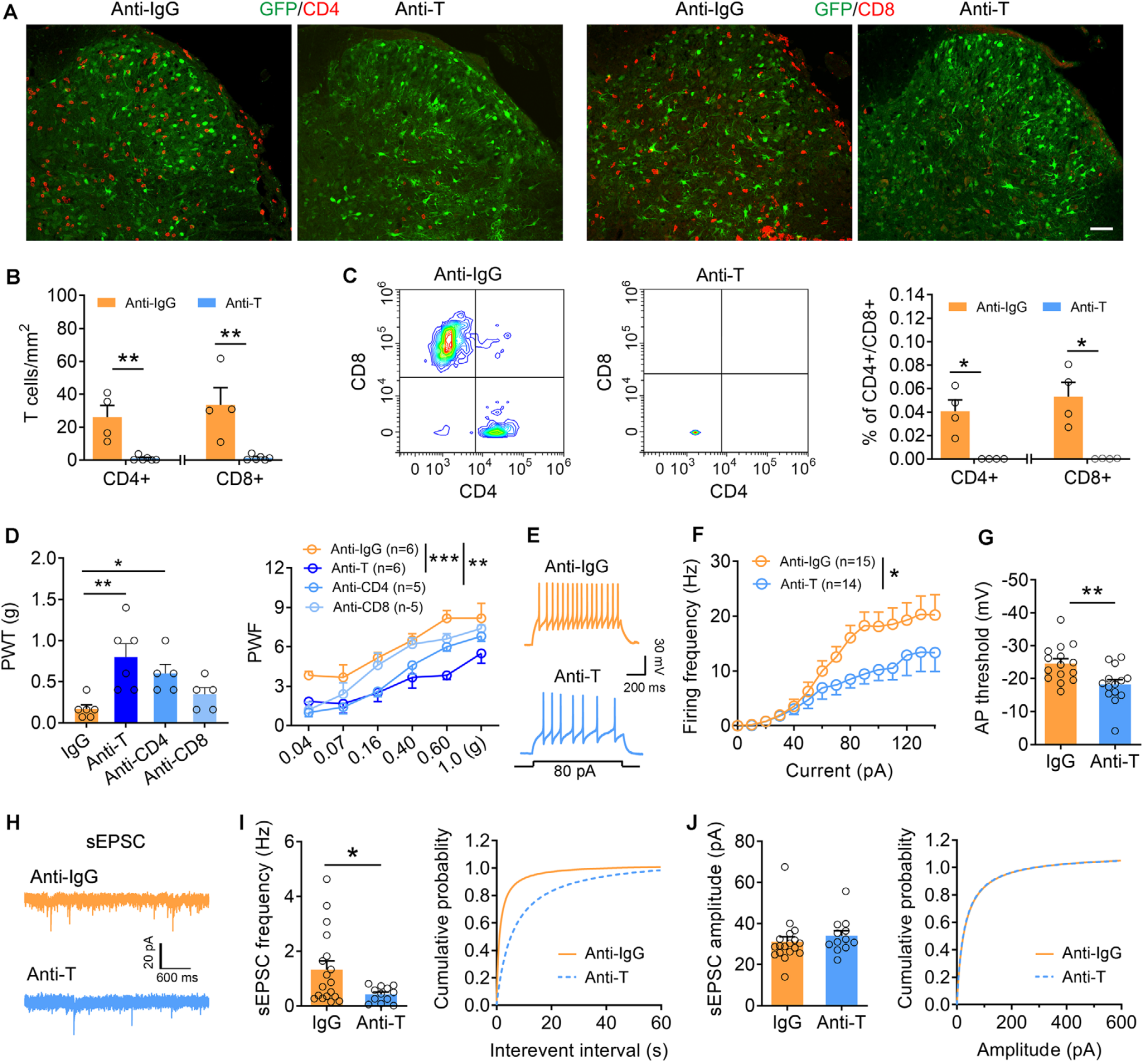
T cell depletion rescues mechanical allodynia and spinal SOM+ neuronal hyperexcitability induced by astrocyte‐specific SHP1 deficiency. (A, B) Representative immunofluorescence images (A) and quantification (B) showing reduced CD4^+^ and CD8^+^ T cell infiltration in the spinal dorsal horn following T cell depletion. Scale bar, 50 µm. n = 4. (C) Representative flow cytometry plots and quantification confirming reduced CD4^+^ and CD8^+^ T cell populations after T cell depletion. n = 4. (D) CD4 and CD8 neutralizing antibody cocktail (anti‐T) or CD4, but not CD8, neutralizing antibody alone both ameliorated Ptpn6 cKO‐induced mechanical allodynia. n = 5–6. (E) Representative traces of APs in spinal SOM^+^ neurons in different treatment groups. (F, G) Quantification of AP firing frequency (F) and threshold (G) in spinal SOM^+^ neurons from different treatment groups. n = 14–15 cells from four mice per group. (H) Representative traces of sEPSCs in SOM^+^ neurons from different treatment groups. (I and J) Quantification of frequency (I) and amplitude (J) of sEPSC and corresponding cumulative distribution in SOM^+^ neurons from different treatment groups. n = 12–18 cells from four mice per group. All data are presented as mean ± SEM. ^*^ *p* < 0.05, ^**^ *p* < 0.01.

**FIGURE 5:**
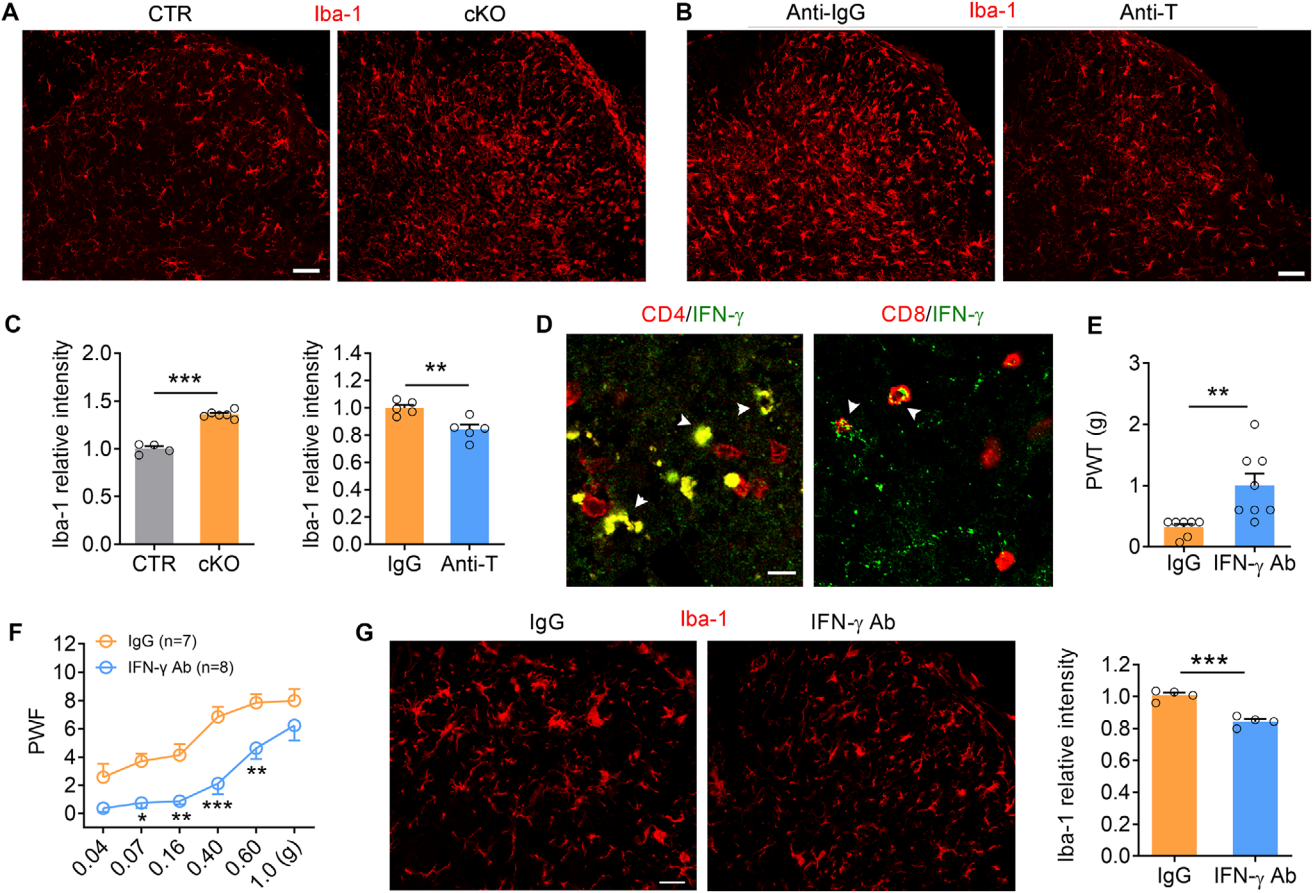
T cell‐derived IFN‐γ participates in mediating spinal microglial activation induced by astrocyte‐specific SHP1 deficiency. (A) Representative images of Iba‐1 fluorescence intensity in the spinal dorsal horn in control and cKO mice. Scale bar, 50 µm. (B) Representative images of spinal Iba‐1 fluorescence intensity after T cell depletion. Scale bar, 50 µm. (C) Quantification analysis of Iba‐1 immunofluorescence intensity in different treatment groups. n = 5. (D) Immunofluorescence staining showing co‐localization of IFN‐γ with CD4^+^ and CD8^+^ T cells in cKO mice. Scale bar, 15 µm. (E and F) Intrathecal administration of IFN‐γ neutralizing antibody significantly alleviates mechanical allodynia induced by astrocyte‐specific SHP1 deficiency, as measured by PWT (E) and PWF (F). n = 7–8. (G) Representative images and quantification of Iba‐1 immunofluorescence intensity with intrathecal injection of IgG or IFN‐γ neutralization. Scale bar, 30 µm. n = 4. All data are presented as mean ± SEM. ^*^ *p* < 0.05, ^**^ *p* < 0.01, ^***^ *p* < 0.001.

### 2.5 Astrocytic CXCL10 Mediates T Lymphocyte Infiltration

Analysis of astrocytic DEGs revealed upregulation of several secreted proteins, particularly chemokines, in the SDH of *Ptpn6* cKO mice. Among these, C‐X‐C motif chemokine ligand 10 (CXCL10) was significantly elevated (Figure 6A). CXCL10 plays a critical role in promoting T cell infiltration under inflammatory conditions and in the pathogenesis of Alzheimer's disease [25, 26]. Immunofluorescence double labeling, Western blotting, and enzyme‐linked immunosorbent assay (ELISA) confirmed a significant increase in CXCL10 expression in spinal astrocytes following *Ptpn6* deletion (Figure 6B–D). To investigate whether CXCL10 mediates T cell infiltration into the spinal cord, we injected an AAV driven by the gfaABC1D promoter expressing both Cre recombinase and *Cxcl10*‐shRNA into the SDH of *Ptpn6* ^flox/flox^ mice to simultaneously knock down astrocytic *Ptpn6* and *Cxcl10* (Figure 6E,F). Astrocyte‐specific *Cxcl10* knockdown significantly reduced the numbers of CD4^+^ and CD8^+^ T lymphocytes in the SDH (Figure 6G–J). Correspondingly, *Ptpn6* cKO‐induced mechanical allodynia was significantly alleviated by *Cxcl10* knockdown (Figure 6K, L).

**FIGURE 6:**
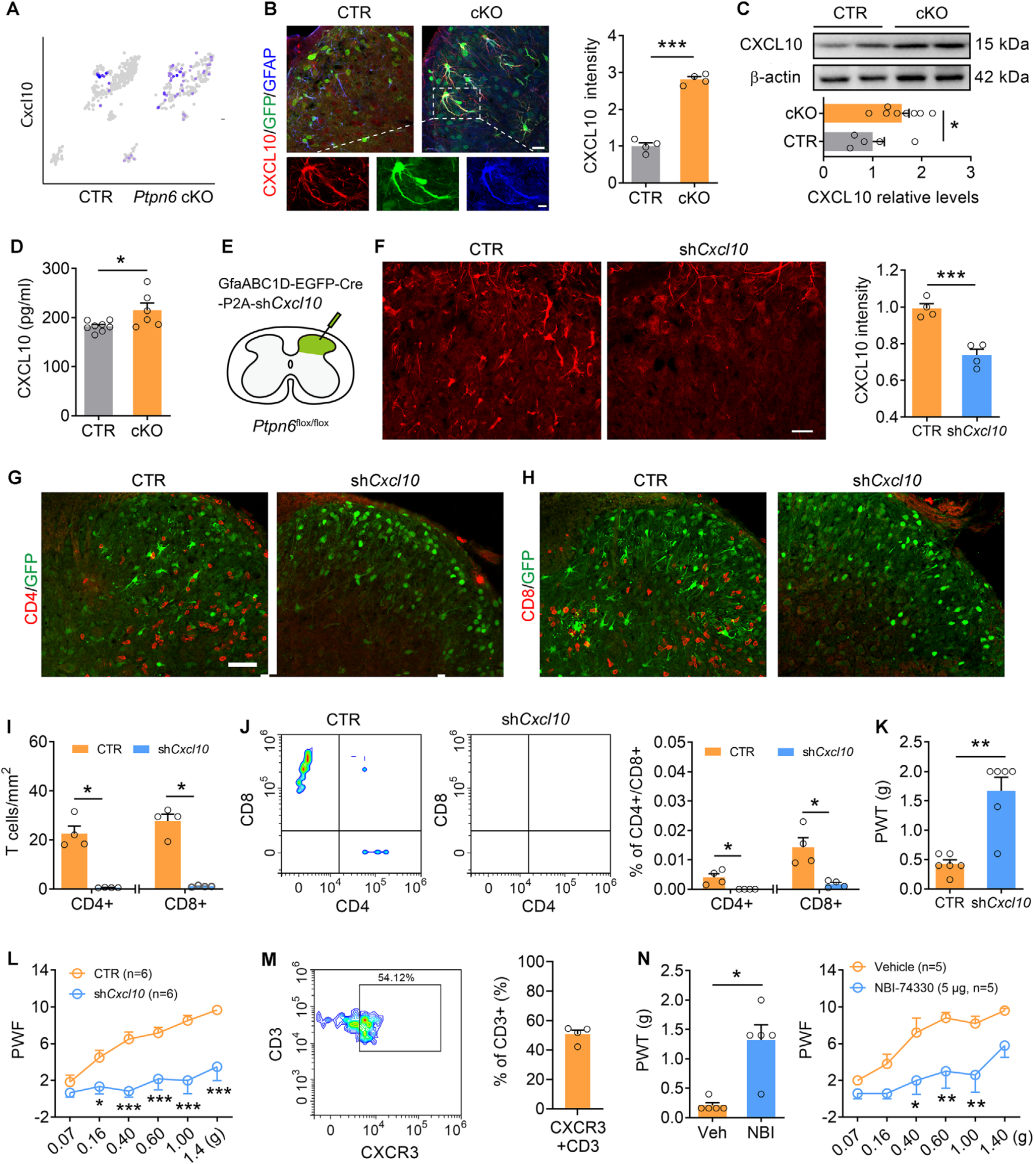
Astrocyte‐specific SHP1 deficiency promotes T cell infiltration into the spinal dorsal horn via CXCL10 signaling. (A) Single‐nucleus RNA sequencing indicates upregulation of CXCL10. (B) Immunofluorescence staining showing increased CXCL10 expression in the spinal dorsal horn of cKO mice. Scale bar, 20 µm. n = 5. (C) Western blot analysis confirming upregulation of CXCL10 protein levels in the spinal dorsal horn of cKO mice. n = 5–8. (D) ELISA showing elevated CXCL10 levels in spinal cord tissue lysates of cKO mice. n = 6–8. (E) Schematic of the strategy for *Cxcl10* knockdown via viral infection of *Cxcl10*‐shRNA on spinal astrocytes in *Ptpn6* ^flox/flox^ mice. (F) Immunofluorescence staining showing the knockdown efficiency of CXCL10. Scale bar, 20 µm. n = 4. (G and H) Representative immunofluorescence images showing reduced CD4^+^ (G) and CD8^+^ (H) T cell infiltration in the spinal dorsal horn following *Cxcl10* knockdown. Scale bar, 50 µm. (I) Quantification of CD4^+^ and CD8^+^ T cell numbers after *Cxcl10* knockdown. n = 4. (J) Representative flow cytometry plots and quantification of T cell populations. n = 4. (K and L) Knockdown of *Cxcl10* significantly reverses mechanical allodynia induced by astrocytic‐specific SHP1 deficiency. n = 6. (M) Proportion of CXCR3^+^ cells among CD3^+^ T lymphocytes in the spinal dorsal horn. n = 4. (N) Blockade of CXCR3 by intrathecal NBI‐74330 (CXCR3 antagonist) alleviates mechanical allodynia induced by astrocytic‐specific SHP1 deficiency. n = 5. All data are presented as mean ± SEM. ^*^ *p* < 0.05, ^**^ *p* < 0.01, ^***^ *p* < 0.001.

CXCR3 is the cognate receptor mediating CXCL10 signaling [26]. Flow cytometry revealed that the infiltrating T lymphocytes expressed CXCR3 (Figure 6M), suggesting that astrocyte‐derived CXCL10 recruits T lymphocytes via CXCL10–CXCR3 interactions. Intrathecal administration of the CXCR3 antagonist NBI‐74330 (5 µg) significantly attenuated *Ptpn6* cKO‐induced mechanical allodynia (Figure 6N). These findings indicate that the CXCL10/CXCR3 axis plays a pivotal role in mediating T lymphocyte infiltration into the spinal cord and the development of mechanical allodynia in *Ptpn6* cKO mice.

### 2.6 STAT1‐CXCL10‐CXCR3 Axis Mediates T Lymphocyte Infiltration

To elucidate the mechanism by which *Ptpn6* deficiency upregulates CXCL10 expression, we identified 46 transcription factor (TF) modules, among which members of the STAT family (*Stat1*, *Stat2*, and *Stat3*), *Irf2*, and *Nfkb1* exhibited high AuCell scores (Figure 7A). Notably, the STAT family exhibited a more pronounced upregulation following *Ptpn6* deletion compared to *Irf2* and *Nfkb1* (Figure 7B). Integration of STAT family transcription factor networks from the TRRUST database with astrocytic DEGs revealed that Stat1 directly regulates *Cxcl10* transcription (Figure 7C). Consistent with previous studies implicating STAT1 as a key inducer of CXCL10 [27, 28]. *Ptpn6* deficiency resulted in a significant increase in phosphorylated STAT1 (pSTAT1) levels (Figure 7D,E), indicating that SHP1 modulates STAT1 phosphorylation. Co‐IP assays using primary cultured spinal astroglial cells and HEK293T cells coexpressing SHP1 and STAT1 demonstrated a direct interaction between SHP1 and STAT1 (Figure 7F and Figure S6A). As shown above, more than 80% of astrocytes expressed SHP1. We further demonstrated that STAT1 was expressed in nearly all astrocytes (Figure S6B), consistent with a SHP1‐STAT1 interaction within spinal astrocytes. Inhibition of STAT1 by intrathecal administration of fludarabine (Flud, 10 µg), a selective STAT1 inhibitor, effectively prevented *Ptpn6* cKO‐induced upregulation of STAT1 and CXCL10 (Figure 7G and Figure S6C,D). Correspondingly, Flud treatment significantly reversed *Ptpn6* cKO‐induced mechanical allodynia (Figure S6E). These findings indicate that SHP1 regulates CXCL10 expression via the STAT1‐CXCL10 axis in spinal astrocytes. Consistently, CUT&Tag analysis of SDH tissue from control and *Ptpn6* cKO mice revealed that *Ptpn6* deficiency significantly increased STAT1 chromatin binding near promoter regions (10.89% vs. 44.3%) and overall STAT1 occupancy (Figure S7A–C). Notably, the promoter and distal intergenic regions of *Cxcl10* showed pronounced enrichment peaks, indicating that STAT1 likely directly regulates *Cxcl10* transcription (Figure S7D). Furthermore, genes associated with STAT1‐specific binding sites in *Ptpn6* cKO mice were significantly enriched in immune‐related pathways (Figure S7E). These findings suggest that *Ptpn6* deficiency impairs the dephosphorylation of pSTAT1, leading to its nuclear accumulation and enhanced binding to promoter regions, initiating the transcription of downstream immune‐regulatory genes, such as *Cxcl10*.

**FIGURE 7:**
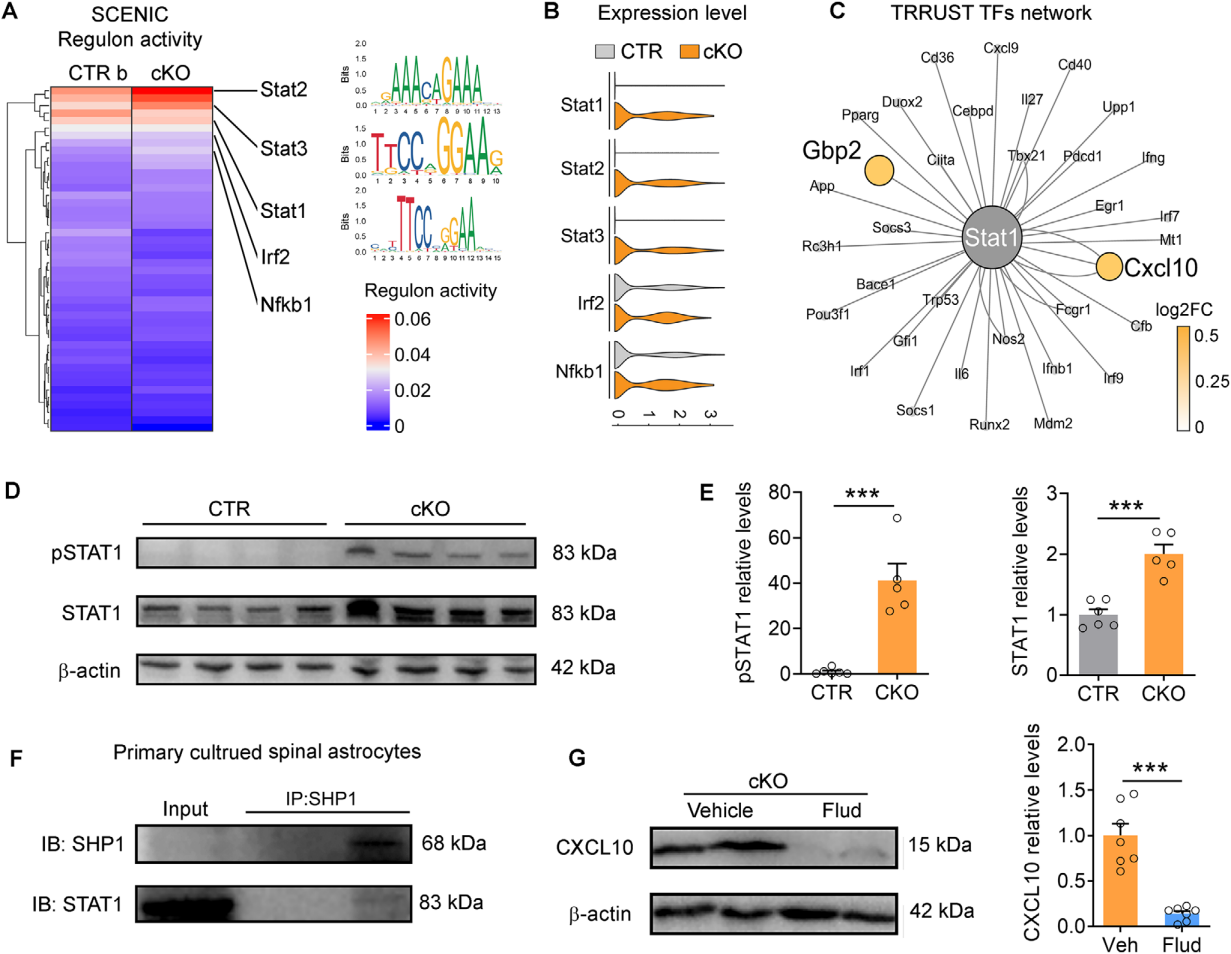
STAT1 transcriptionally upregulates CXCL10 expression in astrocyte‐specific SHP1 deficiency mice. (A) SCENIC analysis predicting elevated transcriptional activity of STAT1 in spinal astrocytes after SHP1 deletion. (B) Expression levels of transcription factors predicted to be highly active in cKO mice. (C) Regulatory network showing connections between STAT family transcription factors (gray nodes) and differentially expressed genes (yellow nodes) identified from single‐nucleus RNA sequencing data based on the TRRUST database. (D, E) Western blot analysis showing increased expression levels of STAT1 and phosphorylated STAT1 (pSTAT1). n = 5–6. (F) Co‐immunoprecipitation (Co‐IP) showing interaction between SHP1 and STAT1 in primary cultured spinal astrocytes. (G) Western blot analysis showing downregulation of CXCL10 expression in fludarabine (Flud, 10 µg, and STAT1 inhibitor) treatment mice. n = 7. All data are presented as mean ± SEM. ^***^ *p* < 0.001.

### 2.7 Spared Nerve Injury Leads to SHP1 Downregulation and Subsequent Alterations

To further elucidate the role of astrocytic SHP1 in spinal nociceptive modulation under neuropathic pain conditions, we examined the expression levels of SHP1 and CXCL10 in the SDH of the SNI mouse model. As expected, western blot analysis revealed that SHP1 was significantly downregulated as early as 1 week post‐SNI, concurrent with increased CXCL10 expression (Figure 8A,B). Differently, significant BBB leakage did not appear until 2 weeks after SNI (Figure 8C). Unlike astrocyte‐specific SHP1 deletion, which caused robust T cell infiltration, SNI induced only minimal T cell infiltration into the SDH. No T cells were detected at 1 week post‐SNI; sporadic infiltration (1–3 cells) appeared at 2 weeks, and 4–8 cells were consistently observed by 3 weeks (Figure 8D,E). These findings suggest that during the development of neuropathic pain, spinal SHP1 and CXCL10 expression levels, BBB leakage, and T cell infiltration are disease course‐dependent. Given the reduction of SHP1 in SNI mice, we hypothesized that restoring SHP1 expression in SDH astrocytes might alleviate SNI‐induced mechanical allodynia and attenuate the activation of astrocytes. To test this hypothesis, we injected AAV‐DIO‐*Ptpn6* into the SDH of Aldh1l1^CreERT2^ mice to overexpress SHP1 selectively in astrocytes (Figure 8F–H). SHP1 overexpression significantly reversed SNI‐induced mechanical allodynia (Figure 8I). The activation of astrocytes induced by SNI was also significantly suppressed (Figure 8J). These findings emphasize a pivotal role for astrocytic SHP1 in modulating spinal nociceptive processing.

**FIGURE 8:**
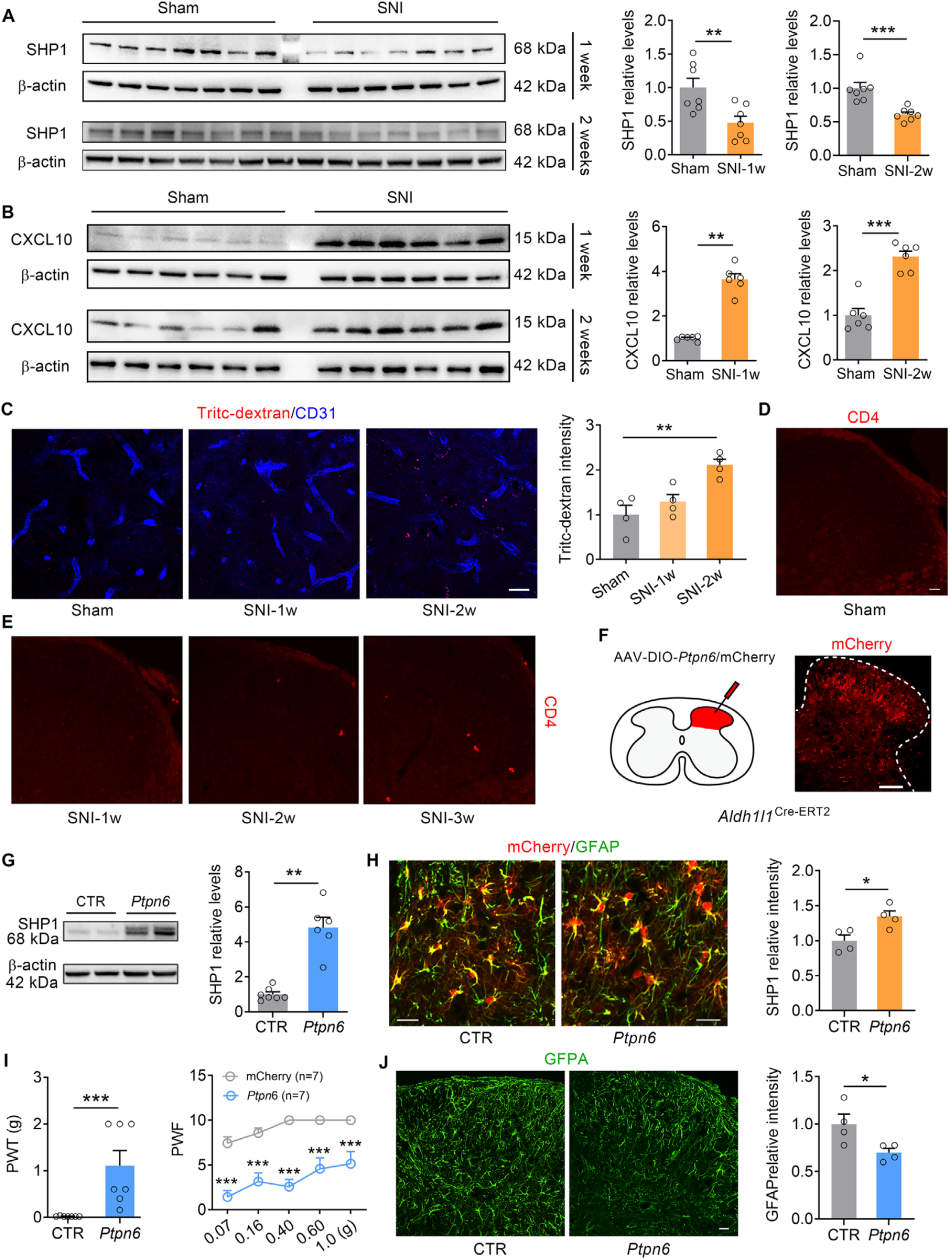
Astrocytic overexpression of SHP1 alleviates SNI‐induced mechanical allodynia and astrocytic activation. (A, B) Western blot analysis showing the expression levels of SHP1 (A) and CXCL10 (B) in the SDH of sham and SNI mice at 1 and 2 weeks post‐surgery. n = 6–7. (C) Immunohistochemistry staining showing dextran leakage in the spinal dorsal horn parenchyma in sham and SNI mice at 1 and 2 weeks post‐surgery. Scale bar, 20 µm. n = 4. (D, E) Representative immunofluorescence images of T‐cell infiltration in the SDH of sham (D) and SNI (E) mice at 1–3 weeks post‐surgery. Scale bar, 20 µm. (F) Schematic and photograph showing *Ptpn6* viral infection in the SDH of Aldh1l1^CreERT2^ mice to overexpress astrocyte‐specific SHP1 Scale bar, 100 µm. (G) Western blot analysis showing the efficiency of SHP1 overexpression in the SDH. n = 6‐7. (H) Representative immunofluorescence images and quantification of SHP1 overexpression in SDH astrocytes. n = 4. Scale bar, 20 µm. (I) Overexpression of SHP1 in spinal astrocytes rescues SNI‐induced mechanical allodynia. n = 7. (J) Representative immunofluorescence images and quantification showing that SHP1 overexpression attenuates SNI‐induced astrocyte activation. n = 4. Scale bar, 20 µm. All data are presented as mean ± SEM. ^*^ *p* < 0.05, ^**^ *p* < 0.01, ^***^ *p* < 0.001.

## 3 Discussion

SHP1 is a multifunctional protein tyrosine phosphatase broadly expressed in peripheral immune cells, where its diverse substrates and dephosphorylation activity have been implicated in immune regulation and tumorigenesis [29, 30]. We extend the functional significance of SHP1 to the central nervous system by demonstrating its critical involvement in modulating spinal neuroinflammation and nociception. In particular, we identify a pivotal role of spinal astrocytic SHP1 in regulating nociceptive hypersensitivity. Loss of SHP1 in spinal astrocytes elicited a tripartite neuroimmune response characterized by (1) reactive astrocytic morphology, (2) T lymphocyte infiltration into the SDH, and (3) microglial activation. The dynamic interplay between reactive astrocytes, infiltrating T lymphocytes, and activated microglia establishes a pronociceptive chemokine–cytokine network within the SDH, amplifying neuronal excitability and behavioral hypersensitivity through intricate signaling cascades (Figure 9). Importantly, we observed a significant downregulation of SHP1 in the SDH as early as 1 week post‐SNI, suggesting its critical role in the early stage of neuropathic pain development. Overexpression of SHP1 in spinal astrocytes reversed SNI‐induced astrocytic activation and mechanical allodynia. Supporting this, reduced SHP1 levels and activity have been reported in human neuroinflammatory diseases, including multiple sclerosis with a prominent pain phenotype [31, 32]. These findings suggest that SHP1 may represent a potential therapeutic target for pain‐related neurological disorders.

**FIGURE 9:**
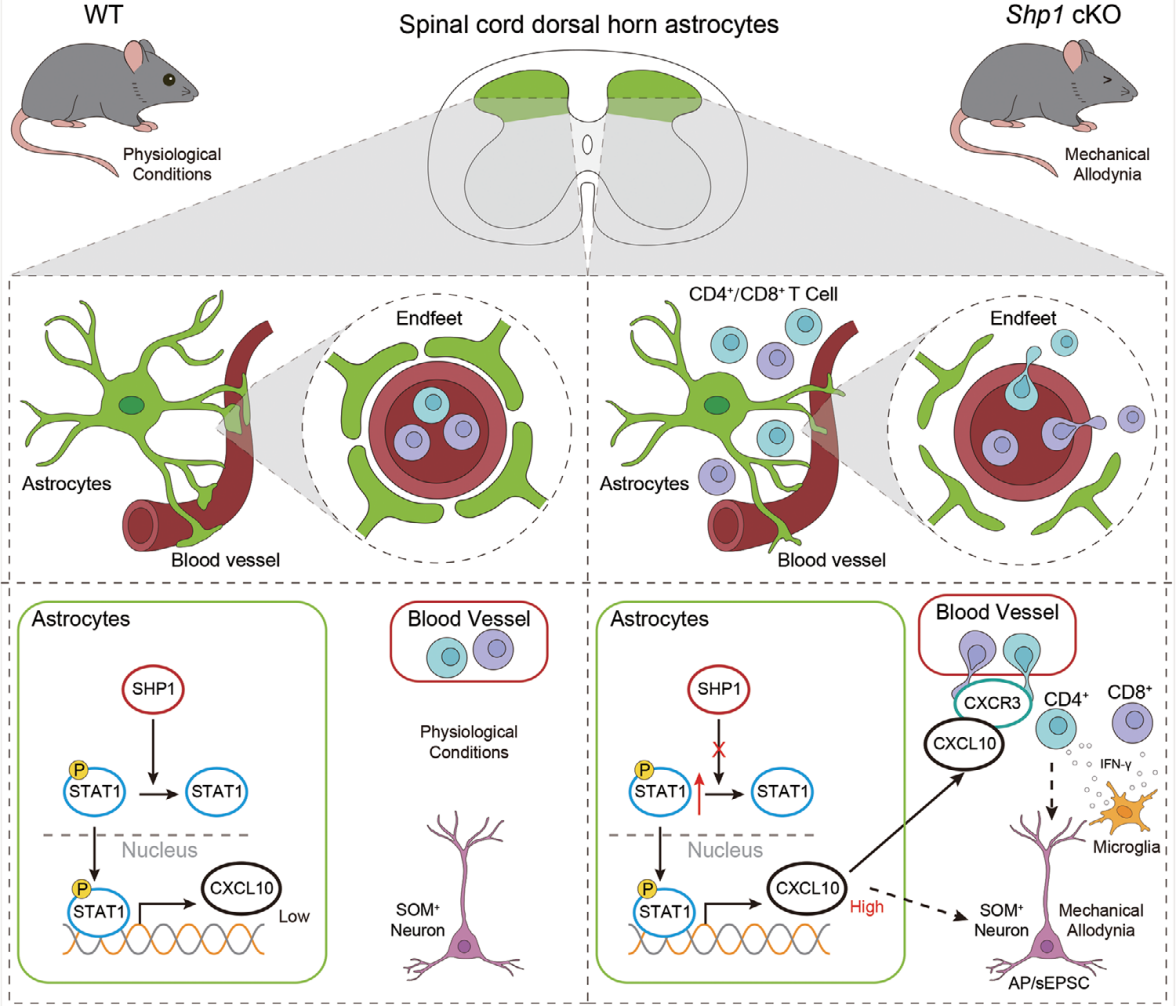
Schematic illustration of mechanisms for astrocytic SHP1 in spinal dorsal horn physiology and pathology. Astrocyte‐specific SHP1 deletion disrupts astrocyte–vascular interactions and compromises BBB integrity while enhancing STAT1‐dependent CXCL10 expression. These changes synergistically promote peripheral CD4^+^ and CD8^+^ T‐cell infiltration into the SDH. The infiltrating T cells secrete IFN‐γ, which in turn activates microglia. Together, these events contribute to hyperexcitability of spinal dorsal horn SOM^+^ neurons and ultimately induce nociceptive hypersensitivity.

snRNA‐seq analysis revealed that astrocyte‐specific *Ptpn6* deletion drove robust CD4^+^ T and CD8^+^ T lymphocyte infiltration into the SDH parenchyma, suggesting disruption of barrier function. The CNS is an immune‐privileged environment safeguarded by the BBB, which separates the CNS parenchyma from the peripheral circulation and stringently limits the transmigration of blood‐borne cells, proteins, and other macromolecules from the bloodstream into the CNS [33]. Astrocytic endfeet ensheathe nearly the entire cerebrovascular surface and constitute an important component of the BBB [34, 35]. Ablation of astrocytes leads to abnormal expression and function of the tight junction proteins and permits the extravasation of large plasma proteins, such as fibrinogen, into the brain parenchyma [36]. Similarly, astrocyte‐specific deletion of the tight junction protein Cldn4 exacerbates leukocyte and humoral factor infiltration into the parenchyma [37], emphasizing the essential role of astrocytes in maintaining BBB integrity and regulating immune cell and molecular influx [36]. Conversely, the present study observed that astrocyte‐specific SHP1 deletion markedly increased the expression of tight junction proteins (Occludin, Claudin, and ZO‐1), contrasting with the typical finding that BBB disruption is accompanied by their reduction [38, 39]. We speculate that SHP1 deficiency may result in remodeling of tight junction proteins rather than a simple downregulation. Previous studies have also reported that the upregulation of BBB‐associated tight junction proteins may represent a compensatory remodeling response during BBB impairment [40, 41]. Notably, our current findings demonstrate that inhibition of SHP1 activity or knockdown of *Ptpn6* significantly increased polymerized F‐actin in astrocytes, implicating SHP1 in the regulation of astrocytic cytoskeletal dynamics, and BBB stability. Consistent with BBB disruption, *Ptpn6* cKO mice exhibited pronounced reactive morphological alterations in astrocytes, most notably a reduction in vascular endfeet attachment. This phenotype suggests compromised BBB integrity, which may result either from direct interaction of SHP1 with F‐actin or from tVAV3‐mediated F‐actin remodeling. In addition, transcriptomic analysis revealed significant downregulation of prostaglandin D_2_ synthase (*Ptgds*) and upregulation of complement‐related genes, including *C1qa*, *C1qb*, and *C4*, in spinal astrocytes lacking SHP1, which may collectively contribute to BBB leakage and T lymphocyte infiltration. PTGDS catalyzes prostaglandin D2 (PGD2) production, which suppresses inflammatory cell infiltration via DP1 receptor signaling; its deficiency promotes vascular permeability and enhances inflammatory cytokine expression [42, 43]. In parallel, complement activation has been implicated in BBB disruption. Activation of complement component 4 (C4) generates C4‐anaphylatoxin, which increases BBB permeability, whereas loss or inhibition of C1q attenuates BBB leakage in ischemic models [44, 45].

Beyond BBB dysfunction, astrocyte‐derived chemokines facilitate inflammatory cell infiltration. For instance, in a mouse model of depression, astrocytic CCL5 mediates CCR5^+^ neutrophil infiltration into the hippocampus [46]. In our study, we demonstrated that spinal astrocyte‐derived CXCL10 mediates *Ptpn6* cKO‐induced T lymphocyte infiltration into the SDH through its receptor CXCR3 on T cells. Consistently, a distinct subset of *Cxcl10* ^+^ astrocytes has been identified in inflamed brain tissue, exhibiting perivascular localization, antigen‐presenting capacity, and a gatekeeping function against peripheral immune insults [25]. Consistent with our findings, these *Cxcl10* ^+^ astrocytes exhibit high STAT1 expression, which directly drives *Cxcl10* transcription via promoter binding [25, 27, 28]. Besides its role in chemotaxis‐induced T cell infiltration, CXCL10 has also been implicated in spinal central sensitization, and neuropathic pain regulation [47, 48]. Therefore, elevated CXCL10 may contribute to nociceptive hypersensitivity by both T‐cell recruitment and direct modulation of spinal neuronal activity. However, in our present study, T‐cell depletion significantly attenuated mechanical allodynia, supporting that T cell recruitment represents a major downstream mechanism of astrocytic SHP1 deficiency. These findings define the SHP1–STAT1–CXCL10–CXCR3 signaling axis as a pivotal mechanism promoting T lymphocyte infiltration into the spinal cord.

Interestingly, in SNI neuropathic pain model, downregulation of SHP1, and upregulation of CXCL10 in the SDH were observed much earlier than detectable BBB leakage and T‐cell infiltration, indicating that dysregulation of the SHP1–STAT1–CXCL10–CXCR3 pathway may represent an early event in the development of neuropathic pain. In the astrocytic SHP1 knockout model, however, BBB dysfunction and CXCL10 signaling may be independent events but remain closely interconnected. On one hand, SHP1 deletion‐induced cytoskeletal remodeling and altered astrocyte–vascular interactions may directly impair BBB integrity. On the other hand, persistent STAT signaling activation further promotes CXCL10 production, thereby amplifying neuroinflammation and immune cell recruitment. Previous studies have demonstrated that in inflammatory settings, CXCL10 signaling contributes to BBB disruption and vascular endothelial dysfunction [49, 50]. Conversely, various pro‐inflammatory cytokines that leak into the SDH due to BBB disruption can further facilitate the CXCL10 signaling. This positive feed‐forward loop between CXCL10 upregulation and BBB disruption drives T‐cell infiltration and pain sensitization.

The infiltration of T lymphocytes into the nervous system is a well‐established mechanism underlying neuropathic pain‐like hypersensitivity [51, 52, 53]. We demonstrated that astrocytic *Ptpn6* deficiency‐induced T lymphocyte infiltration into the SDH is essential for mechanical hypersensitivity, as evidenced by the reversal of pain behaviors following T cell depletion. T lymphocytes contribute to pain pathogenesis through the release of pro‐inflammatory cytokines, such as IFN‐γ, interleukin‐17, and interleukin‐23, which sensitize SDH neurons, and promote central sensitization, facilitating the development and maintenance of chronic pain [21, 54]. Interestingly, depletion of CD4^+^ T‐cell, but not CD8^+^ T cells, significantly alleviated mechanical hypersensitivity, suggesting a predominant pro‐nociceptive role for the CD4 T cell subset in this model. This is in line with previous studies demonstrating that infiltrating CD4 T cells, particularly Th1/Th17 subsets, contribute to the maintenance of neuropathic pain through pro‐inflammatory cytokine signaling [55]. In addition, T lymphocytes interact with the local antigen presenting cells, including microglia, to trigger and potentiate microglial activation, further amplifying nociceptive signaling [56, 57]. Consistent with these mechanisms, we observed pronounced microglial activation in the SDH of *Ptpn6* cKO mice. A recent study revealed that CRH from dorsal root ganglion (DRG) elicits nociceptive sensitization by activating CRHR2 on SOM^+^ neurons in the SDH, and that targeted downregulation of this receptor in SOM^+^ neurons relieves neuropathic pain and suppresses spinal astrocytic and microglial activation [20]. Consistent with this, our current study demonstrated that spinal SOM^+^ neurons mediated the nociceptive sensitization induced by astrocyte‐specific SHP1 knockout. Considering that spinal microglia express IFN‐γ receptors and exogenous IFN‐γ induces microglial activation and hyperalgesia, our observation that CD4^+^ and CD8^+^ T cells express IFN‐γ, and that intrathecal IFN‐γ neutralization significantly attenuates *Ptpn6* deficiency‐induced microglial activation and nociceptive hypersensitivity, strongly supports a T cell‐microglia interaction [58]. Moreover, a previous study exhibited that Rag1^−/−^ mice, which lack mature T lymphocytes, exhibited reduced mechanical allodynia and attenuated astrocytic activation following CFA‐induced arthritis, suggesting that spinal astrocytes may serve as key targets of T cell–derived signals [59]. These findings indicate that infiltrated T lymphocytes, activated microglia, and reactive astrocytes form a self‐reinforcing neuroimmune circuit within the SDH that perpetuates pain mediator release, enhances nociceptive neuron excitability, and sustains chronic pain states.

It is well established that neuroimmune modulation of chronic pain exhibits sex dimorphism, with microglia serving as key mediators in males, and T lymphocytes playing a predominant role in females [60, 61]. However, we did not detect significant sex differences. Astrocyte‐specific SHP1 deletion in the SDH, elicited comparable neuroimmune responses in both sexes, including T lymphocyte infiltration, microglial activation, and resulting mechanical hypersensitivity. Similarly, depletion of CX3CR1‐expressing cells has been shown to alleviate neuropathic pain in male and female mice following spinal nerve transection [62]. Moreover, optogenetic activation or chemogenetic inhibition of spinal microglia modulates pathological pain without exhibiting sex‐dependent effects [63, 64].

Our study has several limitations. First, limited by the technical approach, it is challenging to determine the precise temporal sequence of CXCL10 upregualtion, BBB leakage, and T cell infiltration in the SHP1 cKO model. This is because effective SHP1 deletion mediated by viral expression requires approximately 3 weeks, and by which point, all of the above events have already occurred. Future studies using longitudinal imaging and cell type‐specific manipulations will be needed to dissect these sequential neuroimmune events. Second, given that conventional TEM yields only two‐dimensional ultrastructural information, future investigations employing immuno‐EM or three‐dimensional reconstruction approaches could enable a more accurate characterization of astrocyte–vascular interactions and endfoot coverage. Finally, although the SNI mouse model partially recapitulated the spinal neuroinflammation, BBB disruption, and behavioral sensitization induced by astrocyte‐specific SHP1 knockout, it elicited substantially milder T cell infiltration than that observed in the cKO mice. This suggests the complexity of chronic pain mechanisms, and a systematic investigation across distinct chronic pain models, diverse T cell infiltration assays, and various T cell subsets is warranted to further elucidate these observations.

## 4 Material and Methods

### 4.1 Study Design

The objective of this study was to investigate the immune‐restraining role of astrocytic SHP1 in regulating neuroinflammatory amplification and pain modulation. Using conditional astrocyte‐specific SHP1 knockout mice, we first assessed the impact of SHP1 deficiency on astrocyte morphology, blood–spinal cord barrier integrity, and nociceptive behaviors through a combination of behavioral assays and histological analyses. To examine how astrocytic SHP1 loss alters neuroimmune signaling, we integrated molecular and pharmacological approaches with flow cytometry to evaluate STAT1 activation, CXCL10 expression, immune cell infiltration, and microglial activation in the spinal dorsal horn. To determine how these neuroimmune interactions affect neuronal function, whole‐cell patch‐clamp recordings were performed to assess the excitability of spinal SOM^+^ neurons, and chemogenetic approaches were used to manipulate specific components of the astrocyte–neuron network. In addition, single‐nucleus RNA sequencing was performed to characterize transcriptional changes across spinal cord cell populations and to further define the signaling pathways associated with astrocytic SHP1 deficiency. Finally, we used the SNI mouse model to characterize temporal alterations in SHP1 and CXCL10 expression in the SDH, BBB leakage, and T cell infiltration during the development of neuropathic pain, aiming to elucidate the indispensable role of SHP1 in the pathogenesis of chronic pain. Together, these complementary approaches allowed us to delineate a multicellular mechanism whereby loss of astrocytic SHP1 amplifies STAT1–CXCL10–CXCR3 signaling, promotes T lymphocyte infiltration, enhances microglial activation, and ultimately increases spinal nociceptive sensitivity. Animals were randomly assigned to experimental groups, and all experiments and analyses were performed in a blinded manner. Sample sizes and power calculation were based on our previous knowledge and experience with similar experimental models and anticipated biological variables. All experiments were independently replicated, and all data were included in analysis. Data were acquired and analyzed in a blinded manner. Sample sizes (n) and replicates are shown in the figures or figure legends.

### 4.2 Animals

Both male and female mice (8–10 weeks old) were used in the study. The transgenic strains included *Ptpn6* ^flox/flox^ (JAX#21160), *Aldh1l1* ^CreERT2^ mice (kindly provided by Prof. Tianming Gao, Southern Medical University), and *Som* ^flpo^ mice (JAX#028579). *Ptpn6* ^flox/flox^::*Som* ^flpo^ mice were generated by crossing *Ptpn6* ^flox/flox^ mice with *Som* ^flpo^ mice. All mice were maintained under controlled environmental conditions (22°C, 60% relative humidity, and a 12‐h light/dark cycle) with ad libitum access to food and water. No more than four mice were housed per cage. The Committee on the Use of Animal Experiments of Fudan University approved the study protocol (approval no. SYXK 2009‐0082) and followed the policies on the use of laboratory animals issued by the International Association for the Study of Pain (Washington, D.C., USA). Animals were randomly assigned to experimental groups according to sex and body weight. At the end of the experiments, mice were euthanized by carbon dioxide inhalation. Behavioral assessments and electrophysiological recordings were performed by investigators blinded to the experimental groups.

### 4.3 SNI Model Construction

Mice were anesthetized with 2% sodium pentobarbital (50 mg/kg, intraperitoneally), and the right sciatic nerve was exposed. The common peroneal and tibial nerves, two of the three terminal branches of the sciatic nerve, were tightly ligated with 5‐0 polyglycolic‐co‐acid (PGLA) sutures, transected distal to the ligation, and a 2‐mm segment of each nerve was removed. The third branch of the sciatic nerve, the sural nerve, was left intact. Sham‐operated mice underwent identical surgical exposure of the sciatic nerve without ligation or transection. All procedures were performed under sterile conditions.

### 4.4 *von* Frey Test

Mice were habituated to the testing environment for 1 h per day over three consecutive days before assessment. Mechanical allodynia was evaluated by measuring the paw withdrawal thresholds (PWTs) in response to a calibrated series of *von* Frey filaments (0.02–1.4 g; Stoelting Corporation, Wood Dale, IL, USA). Each mouse was placed individually in a transparent chamber positioned on an elevated metal mesh platform, allowing access to the plantar surface of the hind paw. Filaments were applied perpendicularly to the plantar surface in ascending order of force only when the mouse was stationary and standing on all four paws. A valid response was defined as a complete withdrawal of the hind paw from the mesh platform. Each filament was applied for approximately 2 s, repeated 10 times at 30 s intervals. The lowest filament force that elicited a withdrawal response in > 50% of the 10 applications was recorded as the PWT.

### 4.5 AAV Virus and Intra‐SDH Injection

pAAV‐GfaABC1D‐EGFP‐P2A‐Cre‐WPRE (AAV2/9, titer: 1.23 × 10^12^ V.G./ml) and pAAV‐GfaABC1D‐EGFP‐WPRE (AAV2/9, titer: 1.05 × 10^12^ V.G./ml) were purchased from Shanghai Sunbio Medical Biotechnology Co., Ltd. (Shanghai, China). rAAV‐hSyn‐fDIO‐hM4D(Gi)‐mCherry‐WPRE‐hGH polyA, rAAV‐hSyn‐fDIO‐ mCherry‐WPRE‐hGH polyA and rAAV‐EF1α‐DIO‐*Ptpn6*‐P2A‐mCherry‐WPRE‐pA were obtained from BrainVTA Biotechnology Co.,Ltd. (Wuhan, China). pAAV‐GfaABC1D‐EGFP‐P2A‐Cre‐sh*Cxcl10*‐WPRE and pAAV‐GfaABC1D‐EGFP‐P2A‐Cre‐sh*scramble‐*WPRE were constructed from Shanghai Sunbio Medical Biotechnology Co., Ltd. (Shanghai, China). The AVV details were shown in Table S1.

Mice were anesthetized with sodium pentobarbital (50 mg/kg, intraperitoneally). The back skin was incised and overlying connective tissue and muscles were dissected to expose the vertebral column in the lumbar region. The column was secured in a stereotaxic frame (Stoelting Corporation, USA). Under a surgical microscope, the dura was incised in the intervertebral spaces of T13 and L1, as well as L1 and L2 to expose the lumbar enlargement (L3–L5) of the spinal cord. A glass micropipette was inserted into the right SDH at a 45° angle to the spinal surface and advanced 420 µm from the dura (corresponding to an approximate vertical depth of 300 µm) to target the superficial dorsal horn. Two injection sites were established on one side of the SDH from each intervertebral space, with a vertical interval of 220 µm between sites. In total, 400 nL of AAV solution was delivered at each intervertebral level at a rate of 30 nL/min using an air pressure injection system (Nanoliter 2010 Injector, WPI, Sarasota, FL, USA) connected to a glass pipette. The pipette was held in place for 5 min post‐injection to facilitate diffusion before being withdrawn slowly. Following injection, the incision was sutured, and mice were allowed to recover on a heating pad before returning to their home cages. All subsequent experiments were conducted 4 weeks after viral injection to ensure maximal and stable transgene expression. After completion of all experiments, mice were sacrificed to confirm AAV expression localized to the ipsilateral SDH of the L2–L5 spinal cord segments; data from animals without proper viral expression were excluded.

### 4.6 In Vivo T Cell Depletion

For T‐cell depletion experiments, all mice received spinal injection of AAV‐GfaABC1D‐Cre‐EGFP at D0. IgG (100 µg), anti‐CD4 and anti‐CD8 cocktail (each at 100 µg, with a 30‐min interval between the two administrations), anti‐CD4 (100 µg), or anti‐CD8 alone were administered intraperitoneally using insulin syringes. Antibodies were injected every 3 days (on days 0, 3, 6, 9, 12, 15, 18, and 21), for a total of eight injections. Behavioral testing was performed on day 24 followed immediately by tissue collection. Before injection, antibodies were diluted in PBS at the following pH conditions: anti‐CD4, pH 6.5; anti‐CD8, pH 7.0; IgG, pH 7.0.

### 4.7 Intrathecal Injection

Mice were briefly anesthetized using isoflurane (3% for induction and 1.5% for maintenance). Vehicle, drugs, or neutralizing antibodies were delivered into the cerebrospinal fluid (CSF) space between the L5 and L6 vertebrae via a lumbar puncture made by a 30‐gauge needle. Five to ten microliters of solution were injected with a micro‐syringe, with successful spinal puncture confirmed by a brisk tail‐flick. Mice that showed any surgery‐related neurological deficits were excluded from subsequent experiments.

### 4.8 Tail Vein Injection

The tail vein injection was performed using a tail vein injection device and a 32G insulin needle. The mouse was placed in a restrainer, with its head gently secured to allow unobstructed breathing. The tail was extended and secured, and the light source was adjusted to clearly visualize the tail vein. An appropriate injection site was identified, and the area was gently massaged to facilitate vein dilation. The needle was inserted with the bevel facing upward, and the drug was slowly injected.

### 4.9 Chemogenetic Manipulation

For chemogenetic inhibition, we administered the designed clozapine‐*N*‐oxide (CNO, 5 mg/kg, i.p., Sigma–Aldrich, C0832) 30 min before *von* Frey testing, and the testing was completed within 2 h.

### 4.10 Preparation of Spinal Cord Slices and Whole‐Cell Patch‐Clamp Recordings

The L3–L5 lumbar segments of the spinal cord were rapidly removed under deep anesthesia and immediately immersed in ice‐cold cutting artificial cerebrospinal fluid (ACSF), containing (in mm) NMDG 92, KCl 2.5, HEPES 20, NaHCO_3_ 30, Glucose 25, Na‐ascorbate 5, Na‐pyruvate 3, Thiourea 2, MgSO_4_ 10, and CaCl_2_ 0.5, oxygenated with 95% O_2_ and 5% CO_2_ (pH 7.35, 300 to 310 mOsm/liter). Transverse spinal cord slices (280 µm thick) were prepared using a vibrating blade microtome (VT1200S; Leica Microsystems, Nussloch, Germany) and incubated in recording ACSF containing (in mM) NaCl 125, KCl 2.5, NaHCO_3_ 26, NaH_2_PO^4^·2H_2_O 1.25, CaCl_2_ 2, MgCl_2_·6H_2_O 1, and Glucose 25, oxygenated with 95% O_2_ and 5% CO_2_ (pH 7.35, 300 to 310 mOsm/liter) at 32°C for 30 min. After incubation, slices were transferred to a recording chamber and perfused with the oxygenated recording ACSF at a flow rate of 3 mL/min at room temperature (RT).

Whole‐cell patch‐clamp recordings were performed at RT from mCherry‐fluorescent lamina IIo SOM^+^ neurons using a MultiClamp 700B amplifier and a Digidata 1550B digitizer (Molecular Devices, San Jose, CA, USA). Patch pipettes were fabricated from borosilicate glass capillaries (World Precision Instruments, Sarasota, FL, USA) using a horizontal micropipette puller (P‐1000, Sutter Instruments, San Rafael, CA, USA) and had a resistance of 3–8 MΩ when filled with the internal pipette solution. Signals were filtered at 2 kHz, digitized at 10 kHz, and analyzed with Clampfit 10.6 software (Molecular Devices) and Mini Analysis Program (Synaptosoft, Decatur, GA, USA).

For AP recording, the depolarizing currents were injected from –50 to 150 pA in 10 pA steps with 1s duration in current‐clamp mode. Patch pipettes were filled with a K‐gluconate internal solution containing (in mm) KCl 140, MgCl_2_·H_2_O 1, EGTA 5, HEPES 10, ATP·Na_2_ 3, and Na_2_·GTP 0.2, (pH 7.35, 290 to 300 mOsm/liter).

For sEPSC and sIPSC recordings, pipettes were filled with Cs‐methanesulfonate internal solution containing (in mm) Cs‐methanesulfonate 127.5, CsCl 7.5, MgCl_2_·6H_2_O 2.5, EGTA 0.6, HEPES 10, Na_2_Phosphocreatine·4H_2_O 10, Na_2_‐ATP 4, and Na‐GTP 0.4 (pH 7.35, 290 to 300 mOsm/liter). After establishing the whole‐cell configuration, neurons were voltage‐clamped at −70 mV to record sEPSCs and at 0 mV to record sIPSCs.

### 4.11 Immunofluorescence Staining

Mice were deeply anesthetized and transcardially perfused with normal saline followed by 4% paraformaldehyde (PFA, pH 7.4, at 4°C) in 0.1 m phosphate buffer. The L3–L5 spinal cord segments were dissected, postfixed overnight in 4% PFA, and cryoprotected in graded sucrose solutions (10%, 20%, and 30%) at 4°C. Transverse spinal cord sections (14 µm thick) were cut using a cryostat (Leica Microsystems), and mounted onto Superfrost Plus microscope slides Thermo Fisher Scientific, Waltham, MA, USA). Sections were blocked with 10% donkey serum containing 0.3% Triton X‐100 for 2 h at RT and incubated overnight at 4°C with primary antibodies. After three 10 min washes in 0.01 m phosphate‐buffered saline (PBS), sections were incubated with appropriate secondary antibodies for 2 h at RT. For immunocytochemistry, cultured cells grown on poly‐D‐lysine–coated glass coverslips were fixed with 4% PFA for 30 min at RT, washed, blocked for 30 min, and then incubated sequentially with primary and secondary antibodies. Antibody details are provided in Table S1. The specificity of immunostaining was verified by omitting primary antibodies and by the cKO mice. Fluorescence images were acquired using a confocal laser‐scanning microscope (FV3000, Olympus, Tokyo, Japan).

### 4.12 Human Spinal Cord Sections

Human postmortem spinal cord sections were obtained from a donor, a 21‐year‐old healthy female who succumbed to nitrogen gas asphyxiation, through the Shantou University Pathology Center with approval from the Medical Ethics Committee of Shantou University School of Medicine (SUMC‐2025‐025).

### 4.13 Cell Culture

SDH tissue was dissected from neonatal mice, with the meninges carefully removed. The tissue was minced and enzymatically digested with 0.25% trypsin‐ ethylenediaminetetraacetic acid (EDTA) (Gibco, Grand Island, NY, USA) at 37°C for 15 min, followed by gentle mechanical trituration to obtain a single‐cell suspension. Digestion was terminated by adding an equal volume of medium containing fetal bovine serum (FBS). The cell suspension was filtered through a 70‐µm cell strainer to remove tissue debris and centrifuged at 1000 rpm for 5 min. The resulting pellet was resuspended in medium containing Dulbecco's modified Eagle's medium (DMEM)/F12 supplemented with 10% FBS and 1% penicillin‐streptomycin to a final concentration of 5 × 10^5^ cells/mL. The cell suspension (approximately 5 mL per flask) was plated in T25 culture flasks pre‐coated with poly‐D‐lysine (0.1 mg/mL, #P0899, Sigma–Aldrich, St. Louis, MO, USA) for 2 h at 37°C. Cultures were maintained in a humidified CO_2_ incubator (5% CO_2_, 37°C). The culture medium was replaced with a full volume of fresh medium after 24 h and subsequently refreshed at half‐volume every 3 days.

After 2 weeks of culture, astrocytes were purified by orbital shaking at 240 rpm for 4 h at 37°C. The supernatant was discarded, and the remaining adherent cells were washed with Hank's balanced salt solution. The enriched astrocytes were detached with 0.25% trypsin‐EDTA, and the digestion was terminated by adding complete medium. The cell suspension was centrifuged at 1000 rpm for 5 min, resuspended in fresh medium, and seeded into six‐well plates. Experiments were conducted after an additional 2 weeks of culture, once astrocytes reached the desired confluence. The purity of the astrocyte cultures was verified by GFAP immunostaining.

Frozen G422 and HEK‐293 cells were rapidly thawed in a 37°C water bath and transferred to 10 mL of pre‐warmed complete culture medium (DMEM supplemented with 10% FBS and 1% Penicillin‐Streptomycin). The cell suspension was centrifuged at 1000 rpm for 5 min, and the pellet was resuspended in fresh medium. Cells were seeded into six‐well plates and maintained in a humidified incubator at 37°C with 5% CO_2_. When cultures reached approximately 80% confluence, transfection was performed using Lip8000 reagent (Beyotime, Shanghai, China). The transfection mixture consisted of 125 µL serum‐free DMEM, 2.5 µg plasmid DNA, and 4 µL Lip8000 reagent, which was added dropwise to each well. After 16 h, the medium was replaced with fresh complete medium, and cells were harvested 24 h post‐transfection for protein extraction.

### 4.14 Western Blot

Mice were sacrificed with overdose anesthesia, and the L3‐L5 spinal cord segments were rapidly removed. Cultured cells were washed three times with cold PBS before collection. The SDH tissues and cultured cells were homogenized in ice‐cold lysis buffer containing protease and phosphatase inhibitors (Roche Diagnostics, Indianapolis, USA). Protein concentration was measured using a BCA assay (ThermoFisher, 23235). Equal amounts of protein (30 µg) were loaded onto 10% SDS‐PAGE gels (Bio‐Rad, Hercules, CA, USA) for separation and transferred to polyvinylidene difluoride (PVDF) membranes (Millipore, Billerica, MA, USA). Blots were blocked with 5% non‐fat milk for 2 h at RT and then incubated with primary antibodies at 4°C overnight. The blots were further incubated with HRP‐conjugated secondary antibodies (1:5000; Pierce, Rockford, IL, USA) at RT for 2 h. Table S1 lists the antibody sources. All Western blotting was performed three times and consistent results were obtained. Signals were detected by enhanced chemiluminescence (ECL, ThermoFisher, 34095) and captured images with a ChemiDoc XRS System (Bio‐Rad). A Bio‐Rad image analysis system was then used to measure the integrated optical density of the specific bands.

### 4.15 Co‐IP

Cultured cells were washed with ice‐cold PBS and collected for protein extraction, followed by quantification of protein concentration. For each reaction, 2 µg of antibody was incubated with 1000 µg of total cellular protein in lysis buffer (adjusted to a final volume of 500 µL) overnight at 4°C with gentle rotation. The following day, 25 µL of magnetic beads and 175 µL of lysis buffer were added to the mixture and incubated for 1 h at 4°C. The magnetic beads were collected using a magnetic rack, washed sequentially with lysis buffer and distilled deionized water (ddH_2_O), and the immune complexes were eluted with Western blot sample buffer for 10 min. The eluted proteins were subjected to sodium dodecyl sulfate–polyacrylamide gel electrophoresis and immunoblotting as described above.

### 4.16 Transmission Electron Microscope (TEM)

Under deep anesthesia, L3–L5 spinal cord segments were rapidly excised and cut into 1–3 mm^3^ blocks, which were immediately fixed in 2.5% glutaraldehyde at 4°C overnight. After three washes with 0.1 M PBS, the tissues were post‐fixed in 1% osmium tetroxide for 2 h at 4°C. Samples were then rinsed three times with distilled deionized water (ddH_2_O), stained with 2% uranyl acetate overnight, and washed three times again with ddH_2_O. Following dehydration through a graded ethanol series, the tissues were infiltrated with propylene oxide and embedded in Spurr resin. The resin blocks were polymerized at 70°C, sectioned into 70‐nm ultrathin slices using a Leica UC7 ultramicrotome, and imaged using a TEM (JEOL JEM‐1230, Tokyo, Japan). Vessel selection and image analysis were performed in a randomized and blinded manner.

### 4.17 Flow Cytometry

Mice were deeply anesthetized and perfused transcardially with cold PBS. The L3‐L5 SDH segments were harvested, minced, and dissociated into single cells using the Adult Brain Dissociation Kit (130‐107‐677, Miltenyi Biotec, Bergisch Gladbach, Germany) and the gentleMACS Octo Dissociator (Miltenyi Biotec, 130‐096‐427) according to the manufacturer's instructions. The resulting single cells were incubated with Fc receptor blocking reagent and then aliquoted into single‐color and multi‐color staining tubes. Antibodies used for staining are listed in Table S1. Flow cytometric analysis was performed on a CytoFLEX S cytometer (Beckman Coulter, Brea, CA, USA), and data were analyzed with CytExpert software (Beckman Coulter).

### 4.18 CUT&Tag

For CUT&Tag‐seq (Hyperactive Universal CUT&Tag Assay Kit for Illumina, Vazyme, TD901), the basic procedure was performed following previously described methods [65]. Specifically, in that study, the protocol was as follows: cells (1 × 10^5^) were washed, centrifuged, and resuspended in wash buffer. Concanavalin A‐coated magnetic beads were washed twice and then added to the cell suspension, followed by incubation for 10 min at RT. Bead‐bound cells were resuspended in antibody buffer, and 1 µg /1 × 10^5^ cells of STAT1 primary antibody was added. The mixture was incubated overnight at 4°C. After removal of the primary antibody, cells were incubated with 0.5 µg of secondary antibody diluted in Dig‐wash buffer for 1 h at RT. Following three washes with Dig‐wash buffer, cells were incubated with 0.04 µm Tn5 transposase in Dig‐300 buffer for 1 h at RT. After three additional washes, cells were resuspended in tagmentation buffer and incubated for 1 h at 37°C. The reaction was terminated by adding stop solution (0.5 M EDTA, 10% sodium dodecyl sulfate, and 20 mg/mL Proteinase K), followed by overnight incubation at 37°C. DNA was purified by phenol‐chloroform extraction, ethanol precipitation, and RNase A treatment. Polymerase chain reaction (PCR) amplification (12–15 cycles) was performed to enrich Tn5‐fragmented DNA and to incorporate i5/i7 adapters (TruePrep Index Kit V2 for Illumina, Vazyme, TD202), sequencing primers, and sample indices for Illumina sequencing. Amplified products (200–800 bp) were purified with 0.6 ×/1.2 × SPRIselect reagent (Beckman Coulter, B23318) and subjected to Illumina PE150 Nova sequencing.

Raw FastQ files were processed with Trim Galore to remove low‐quality bases and adapter sequences. Cleaned reads were aligned to the mouse reference genome (GRCm39, GENCODE release M31) using Subread (v2.0.1). PCR duplicates, unpaired reads, and low‐quality alignments were removed using Sambamba (v0.6.6) and SAMtools (v1.21). The resulting BAM files were converted into RPKM‐normalized bigWig tracks with deepTools (v3.5.6) and visualized in IGV (v2.16.2) using the “autoscale” option for cross‐sample comparison. Peak calling was performed on the bigWig files using LANCEotron (v1.2.7), and peaks were annotated to the nearest genes with ChIPseeker (v1.40.0).

### 4.19 Single‐Nucleus RNA‐Sequencing (snRNA‐seq)

For snRNA‐seq, approximately 1–2 × 10^4^ nuclei were loaded onto the 10X Genomics Chromium platform to recover 6000–12 000 nuclei. Libraries were constructed using the Chromium Next GEM Single Cell 3' Reagent Kits v3.1, according to the manufacturer's instructions (10x Genomics, CG000205 Rev C). The complementary DNA libraries were purified, quantified using an Agilent 2100 Bioanalyzer, and sequenced on an Illumina NovaSeq 6000 system. Gene expression matrices were generated using Cell Ranger count (10X Genomics, v7.1.0) with the mouse genome GRCm39 as the reference. The expression matrices were corrected for ambient mRNA contamination using SoupX. Low‐quality cells were filtered based on total counts, the number of detected genes, and the percentage of mitochondrial transcripts. Seurat objects were created, and the data were log‐normalized and scaled, with cell cycle and mitochondrial genes regressed out. Principal component analysis (PCA) was performed, and batch effects were corrected using Harmony integration. t‐SNE dimensionality reduction and clustering were performed on the Harmony‐integrated PCs. Gene Ontology (GO) enrichment analysis was conducted using ClusterProfiler.

### 4.20 3D Assessment of Astrocyte Complexity

Three‐dimensional reconstruction of astrocytes within the SDH was performed using Imaris software (version 10.0.0; Oxford Instruments, Abingdon, UK). Astrocyte morphology was analyzed using customized reconstruction parameters. For surface reconstruction, the settings were as follows: surface detail, 5 µm (smooth); thresholding, background subtraction (local contrast); and diameter of the largest sphere fitting into the object, 5 µm. For filament reconstruction, the parameters were: detection of new starting points, largest diameter 8 µm; seed points, 0.3 µm; removal of seed points around starting points, sphere region diameter 10 µm; and seed point threshold, 600. Manual corrections were applied to refine the reconstructions when the automatic segmentation in Imaris was inaccurate. Sholl analysis was performed in filament reconstruction mode to quantify astrocytic branching complexity. All surface and filament parameters were exported to Microsoft Excel files for subsequent data analysis.

### 4.21 Statistical Analysis

All data were summarized as the mean ± standard error of the mean (SEM). The group size is the number of independent values and statistical analysis was done using these independent values. All data from the different groups were verified for normality and homogeneity of variance using Shapiro–Wilk and Brown–Forsythe tests before analysis. No data were transformed or excluded as outliers. Comparisons between two groups were performed using Student's *t*‐test or Mann–Whitney *U* test (nonparametric data). For multiple comparisons, one‐way or two‐way analysis of variance followed by Sidak's *post hoc* test or Kruskal–Wallis H test followed by *post hoc* Dunn's test (nonparametric data) were applied. All analyses were two‐tailed, and *p*‐values <0.05 were considered statistically significant. Statistical analyses were performed using GraphPad Prism 8.0 software (GraphPad Software, San Diego, CA, USA).

## Author Contributions

**Lan‐Xing Yi**, **Lin Yang**, and **Kang‐Li Wang** contributed equally to this work. Conceptualization, **Yu‐Qiu Zhang**. Methodology, Lan‐Xing Yi, Kang‐Li Wang, and Lin Yang. Data curation, Lan‐Xing Yi, Kang‐Li Wang, and Yu‐Qiu Zhang. Investigation, Lan‐Xing Yi, Lin Yang, and Kang‐Li Wang. Validation, **Hui‐Zhu Liu**. Formal analysis, Lan‐Xing Yi, Lin Yang, **Rui‐Ying Chen**, **Xiao Xiao**. Supervision, Yu‐Qiu Zhang. Funding acquisition, Yu‐Qiu Zhang. Project administration, Yu‐Qiu Zhang. Resources, **Min Su**. Writing – original draft, Lan‐Xing Yi, Xiao Xiao. Writing – review and editing, Yu‐Qiu Zhang, Lan‐Xing Yi, and Xiao Xiao.

## Funding

This work was supported by Science and Technology Innovation (STI) 2030‐Major Projects (2021ZD0203200‐05, 2025ZD214900‐2), and National Natural Science Foundation of China (82130032).

## Conflicts of Interest

The authors declare no conflicts of interest.

## Supporting information




**Supporting File 1**: advs76932‐sup‐0001‐SuppMat.pdf.

**Supporting File 2**: advs76932‐sup‐0002‐DataSet.zip.

## Data Availability

All data generated or analyzed during this study are included in this article and its Supplementary information files. The data used in this study can be reasonably requested from the corresponding authors.
