# Supplementary material for: Loss of SHP1 in Spinal Astrocytes Triggers T‐Lymphocyte Infiltration and Nociceptive Hypersensitivity
Source: Adv Sci (Weinh). 2026 Aug 3:e76932. Online ahead of print. doi: 10.1002/advs.76932 (PMC13430923; doi:10.1002/advs.76932)
Supplement: Supplementary file 1 — Supporting File 1: advs76932‐sup‐0001‐SuppMat.pdf. [file ADVS-9999-e76932-s001.pdf]

Supporting Information

Loss of SHP1 in spinal astrocytes triggers T-lymphocyte infiltration and nociceptive hypersensitivity

Lan-xing Yi[#], Lin Yang[#], Kangli Wang[#], Huizhu Liu[1], Ruiying Chen[2], Min Su[3], Xiao Xiao[2]*, Yu-Qiu Zhang[1]*

The PDF file includes:

Figs. S1 to S7

Tables S1

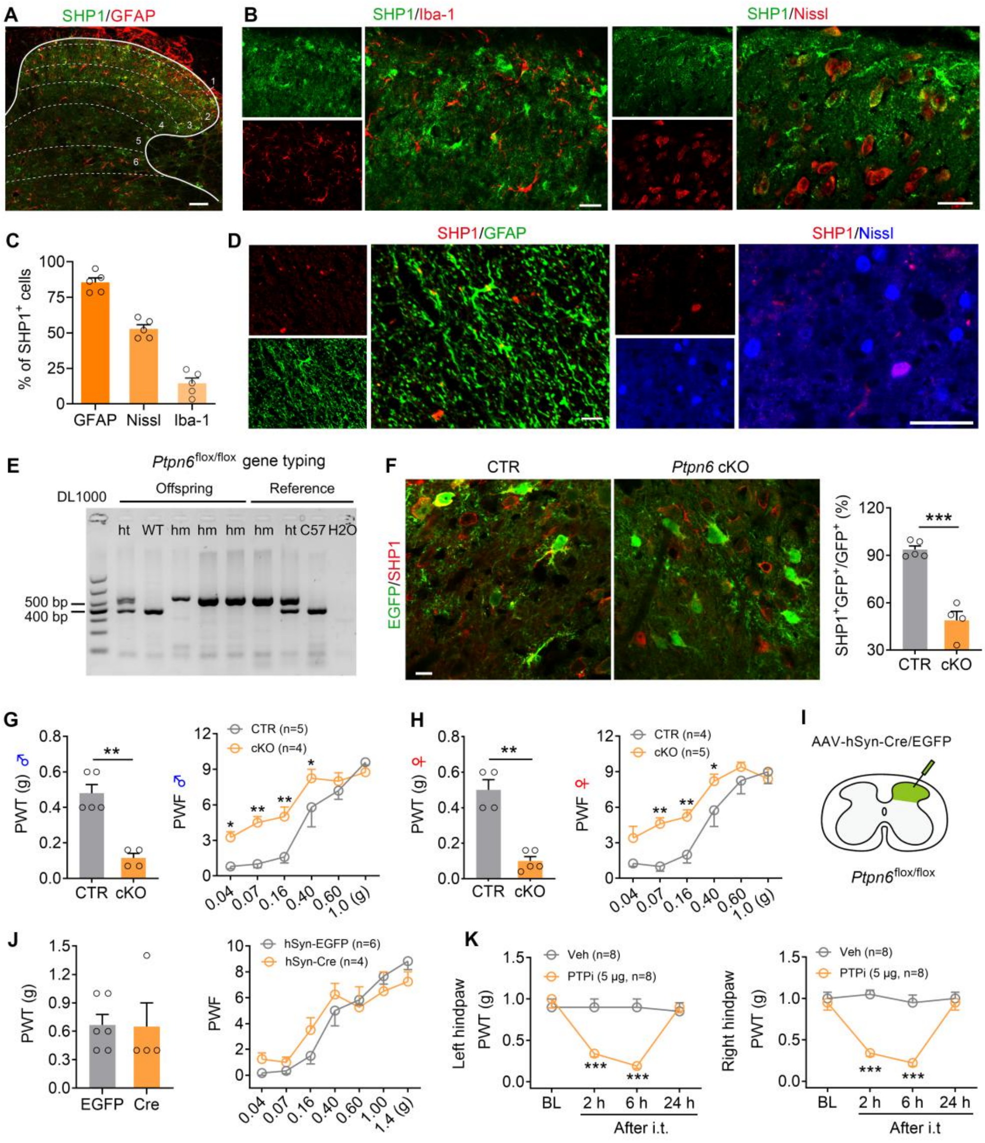

Figure S1. Inactivation of SHP1 induces mechanical allodynia in both male and female mice.

(A) Representative image showing the distribution of SHP1 across laminae of the spinal dorsal horn (SDH) in naive mice. Scale bar, 50 μm. (B) Immunofluorescence co-staining of SHP1 and Iba1 (microglia marker) and Nissl (neuronal marker) in the mouse superficial dorsal horn. Scale bar, 20 μm. (C) Quantitative analysis of the proportional distribution of SHP1 among astrocytes, neurons, and microglia in the SDH. n=5. (D) Representative immunofluorescence co-staining images of SHP1 with GFAP and Nissl in the human SDH. Scale bar, 20 μm. (E) Representative genotyping results of Ptpn6$^{flox/flox}$ mice. (F) Representative images and quantification of SHP1 deletion efficiency following viral infection of GfaABC1D-Cre-mediated recombination in spinal dorsal horn astrocytes of *Ptpn6*$^{flox/flox}$ mice. Scale bar, 10 μm. n = 4–5. (G and H) Paw withdrawal threshold (PWT) and paw withdrawal frequency (PWF) in responses to *von* Frey stimulation of mice treated with astrocytic-specific SHP1 deletion (cKO) and control (CTR) in male (G) and female (H) mice. n=4-5. (I and J) Neuron-specific SHP1 deletion does not alter mice PWT and PWF. n = 4–6. (K) Intrathecal injection of SHP1 inhibitor PTPi (5 μg) produces robust mechanical allodynia in both hind paws. n = 8. Data are presented as mean ± SEM. *$p < 0.05$, **$p < 0.01$, ***$p < 0.001$.

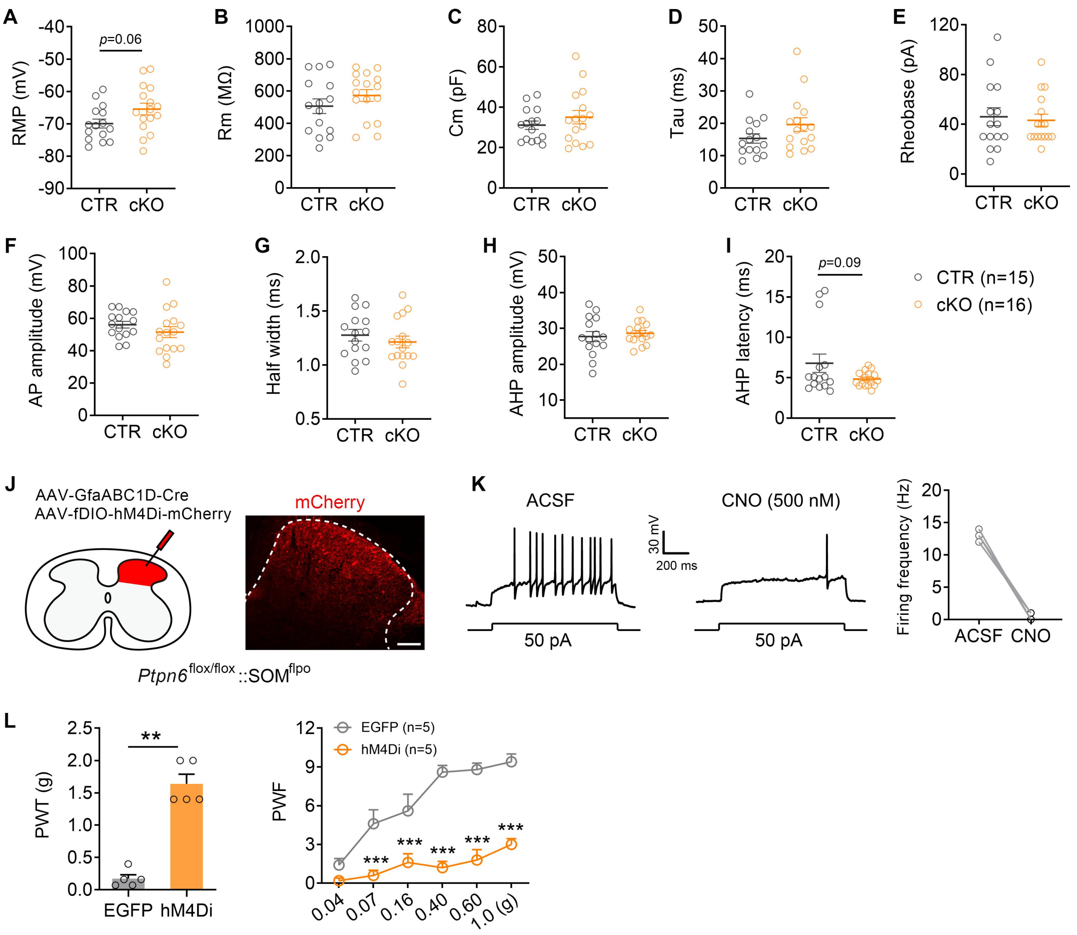

Figure S2. Spinal SOM⁺ neurons contribute to mechanical allodynia induced by astrocytic-specific SHP1 deficiency. (A-I) Resting membrane potential (RMP, A), membrane resistance (Rm, B), membrane capacitance (Cm, C), time constant (Tau, D), Rheobase (E), AP amplitude (F), AP half-width (G), Afterhyperpolarization (AHP) amplitude (H), and Afterhyperpolarization (AHP) latency (I). n=15-16 cells from 4 mice per group. (J) Schematic illustration of the dual-viral infection strategy for astrocyte-specific SHP1 deletion and chemogenetic inhibition of SOM^+ neurons in the spinal dorsal horn. Scale bar, 100 µm. (K) Perfusion of CNO suppresses action potentials (APs) evoked by 50 pA current injections in spinal SOM^+ neurons expressing hM4Di. (L) Chemogenetic inhibition of SOM^+ neurons reversed *Ptpn6* cKO-induced mechanical allodynia as measured by PWT and PWF. n=5.

Data are presented as mean ± SEM. $*p < 0.05$, $**p < 0.01$, $***p < 0.001$.

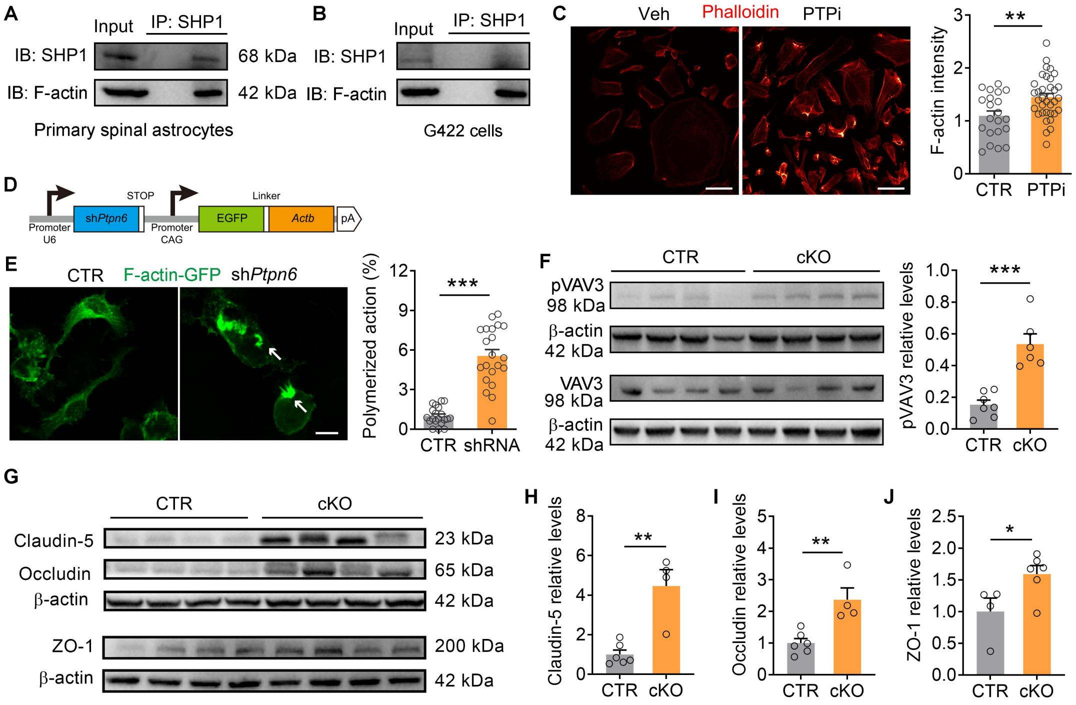

Figure S3. SHP1 regulates astrocytic F-actin polymerization and tight junction proteins. (A) Co-immunoprecipitation (Co-IP) analysis showing interaction between SHP1 and F-actin in primary cultured spinal astrocytes. (B) Co-IP analysis showing interaction between SHP1 and F-actin in G422 glioma cells. (C) Representative phalloidin staining images and quantification of F-actin in primary cultured spinal astrocytes with or without SHP1 inhibitor PTPi treatment. Scale bar, 50 µm. n= 21-35 slices per group. (D) Schematic illustration of the shRNA construct targeting *ptpn6* and co-expression vectors for EGFP and F-actin. (E) Representative images and quantification of F-actin polymerization following knockdown of SHP1 by shRNA in G422 glioma cells. Scale bar: 20 µm. n=20-22 slices per group. (F) Western blot analysis showing changes in VAV3 and phosphorylated VAV3 (pVAV3) expression levels in control and cKO mice. n= 6-7. (G) Representative western blots and quantification of Claudin-5, Occludin, and ZO-1 expression in the SDH of control and cKO mice. n = 4–6.

Data are presented as mean ± SEM. $*p < 0.05$, $**p < 0.01$, $***p < 0.001$.

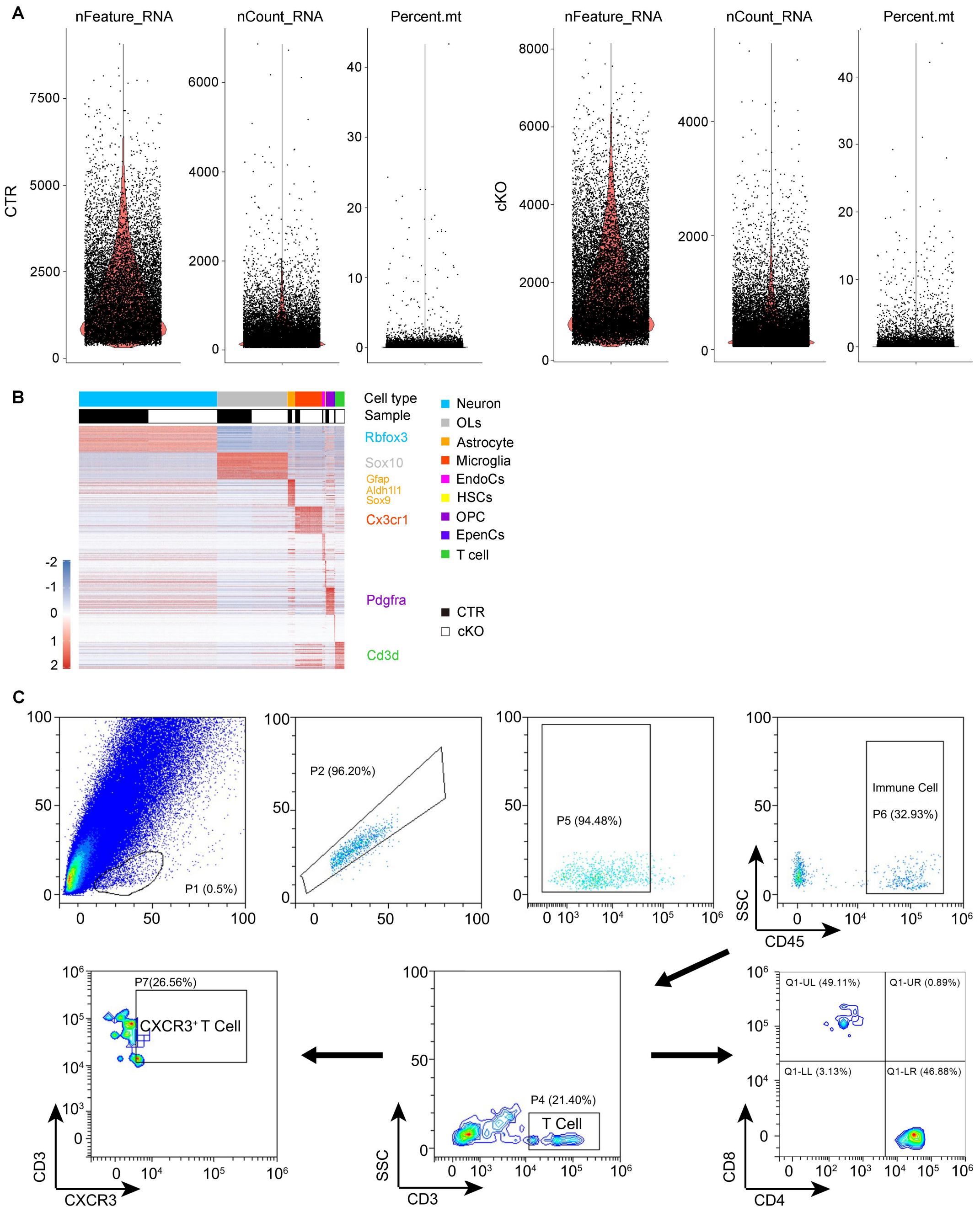

Figure S4. High-quality single-cell RNA-seq data and distinct gene expression profiles across SDH cell types. (A) Quality control metrics of single-cell RNA sequencing datasets from control and cKO spinal dorsal horn tissue. (B) Cell-type specific gene expression profiles illustrating transcriptional identity of spinal dorsal horn populations. (C) Gating strategy used for flow cytometry analysis of spinal immune cell populations.

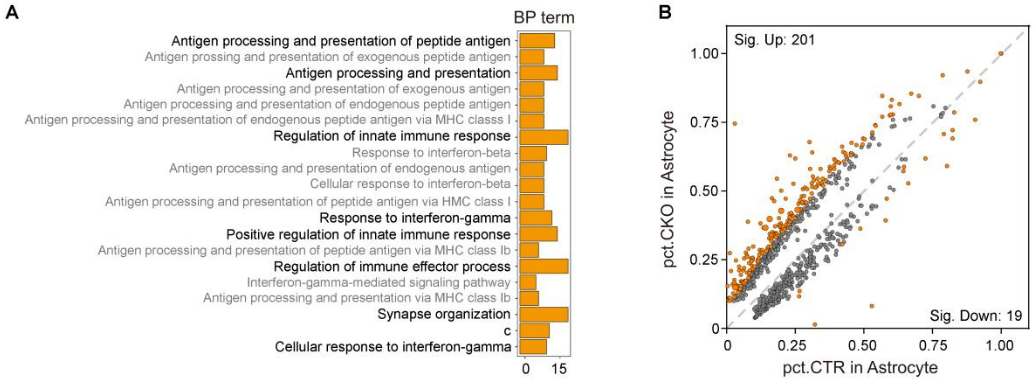

Figure S5. Differentially expressed genes in astrocyte-specific SHP1 deficiency mice are enriched in immune-related functions, related to Figure 4. (A) Gene Ontology (GO) enrichment analysis of biological processes associated with differentially expressed genes (DEGs) in astrocytes following SHP1 deletion. (B) Volcano plot showing DEGs in astrocytes comparing control and cKO groups.

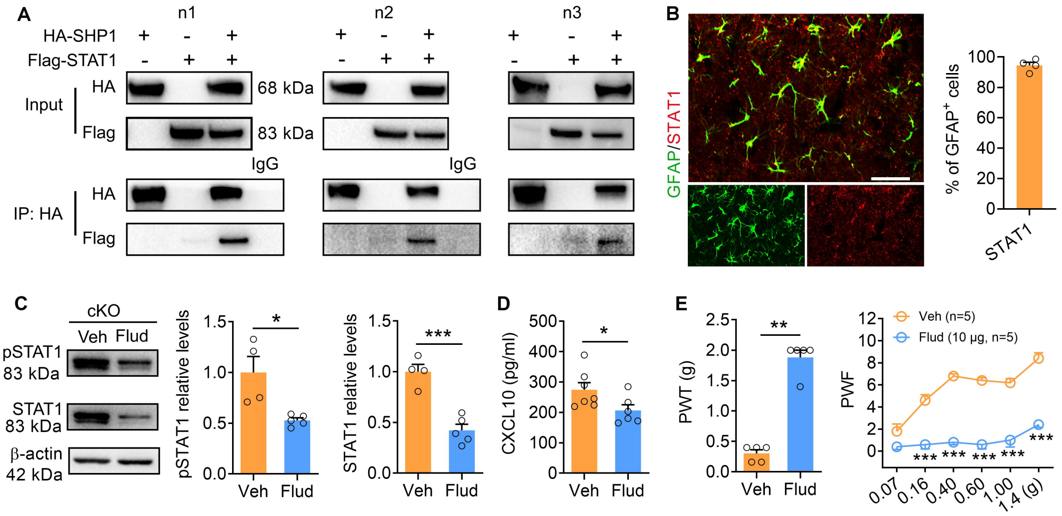

Figure S6. STAT1 contributes to mechanical allodynia induced by astrocyte-specific SHP1 deficiency. (A) Co-IP analysis showing interaction between HA-SHP1 and Flag-STAT1 in transfected HEK-293T cells. (B) Representative immunofluorescence image and quantification of GFAP and STAT1 co-localization in the SDH. n=4. Scale bar, 20 μm. (C) Western blot analysis showing decreased phosphorylated STAT1 (pSTAT1) and total STAT1 following STAT1 inhibitor fludarabine (Flud, 10 μg) application. n=4-5. (D) ELISA showing reduced CXCL10 level following STAT1 inhibitor Flud application. n=6-7. (E) Intrathecal administration of STAT1 inhibitor Flud significantly alleviates mechanical allodynia induced by astrocyte-specific SHP1 deficiency. n=5.

Data are presented as mean ± SEM. $*p < 0.05$, $**p < 0.01$, $***p < 0.001$.

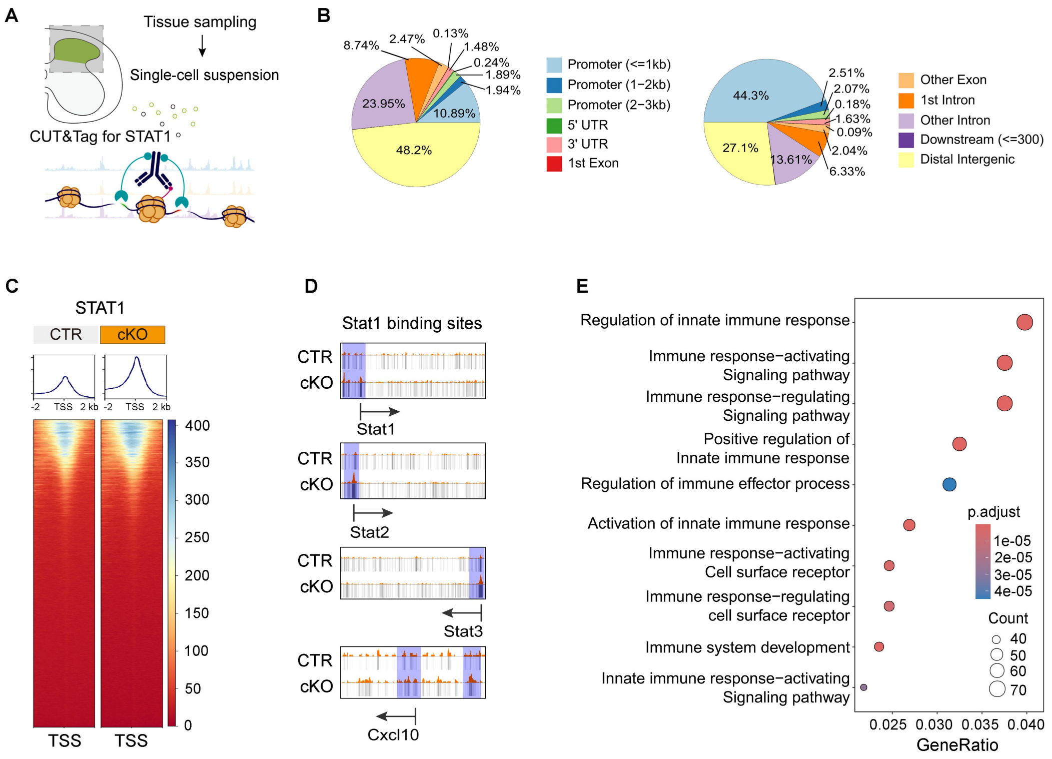

Figure S7. CUT&Tag analysis of SDH tissue from control and *Ptpn6* cKO mice. (A) Schematic diagram of the CUT&Tag experimental workflow used to identify STAT1 binding sites genome-wide. (B) Genomic distribution of STAT1 binding sites across promoter, intronic, intergenic, and other genomic regions under CTR and *Ptpn6* cKO condition. (C) Enrichment profile of STAT1 binding near transcription start sites (TSS) across the genome under CTR and *Ptpn6* cKO condition. (D) Representative genome browser tracks showing STAT1 binding peaks near *Stat* family genes and the *Cxcl10* locus under CTR and *Ptpn6* cKO condition. (E) Functional enrichment analysis of genes associated with STAT1-specific binding sites under *Ptpn6* knockout conditions.

Table S1. The key resources and reagents used in the present study.

**REAGENT or RESOURCE**	**SOURCE**	**IDENTIFIER**
Antibodies		
Rabbit anti Actin (1:10,0000)	ABclonal	CAT#AC026<br>RRID: AB_ 2768234
Rabbit anti CD4 (1:100)	Abcam	CAT#AB183685<br>RRID:AB_2686917
Anti mouse CD4	Bioxcell	CAT#BE0003-1<br>RRID:AB_1107636
Rabbit anti CD8 (1:100)	Abcam	CAT#AB217344<br>RRID: AB_2890649
Anti mouse CD8	Bioxcell	CAT#BE0004-1<br>RRID:AB_1107671
Goat anti CD31 (1:200)	R&D Systems	CAT#AF3628<br>RRID: AB 2161028
Rabbit anti CLAU5 (1:200)	Life Technologies	CAT#34-1600<br>RRID:AB_2533157
Goat anti CXCL10 (1:50)	R&D Systems	CAT#AF-466-NA<br>RRID: AB_2292487
Mouse anti GFAP (1:2000)	Sigma	CAT#G6171<br>RRID: AB_1840893
Rabbit anti GFAP (1:2000)	Abcam	CAT#ab7260<br>RRID: AB_305808
Chicken anti GFP (1:1000)	Aves labs	CAT#GFP-1020<br>RRID: AB_10000240
Goat anti IBA1 (1:500)	Abcam	CAT#ab5076<br>RRID: AB_2224402
Normal Goat IgG	R&D Systems	CAT#AB-108-C<br>RRID:AB_354267

Rabbit anti Occludin (1:200)	Life Technologies	CAT#40-4700<br>RRID:AB_2533468
Rabbit anti pSTAT1 (1:1000)	Cell Signaling	CAT#9167<br>RRID: AB_561284
Rabbit anti pVAV3 (1:1000)	Abcam	CAT#AB109544<br>RRID: AB_10861084
Rabbit anti SHP1 (1:1000)	Proteintech	CAT#24546-1-AP<br>RRID: AB_2879600
Rabbit anti SHP1 (1:500)	GeneTex	CAT#GTX102864<br>RRID: AB_1951541
Rabbit anti STAT1 (1:2000)	Proteintech	CAT#10144-2-AP<br>RRID: AB_2286875
Rabbit anti VAV3 (1:1000)	Abcam	CAT#AB52938<br>RRID: AB_883423
Rabbit anti ZO-1 (1:100)	Life Technologies	CAT#61-7300<br>RRID:AB_2533938
Rat IgG2a isotype control	Bioxcell	CAT#BE0089<br>RRID:AB_1107769
Alexa Fluor® 488 AffiniPure Donkey Anti-Chicken IgY (IgG) (H+L) (1:200)	Jackson	Cat#703-545-155<br>RRID: AB_2340375
Alexa Fluor® 647 AffiniPure Donkey Anti-Rabbit IgG (H+L) (1:200)	Jackson	CAT#A10040<br>RRID: AB_2492288
Donkey anti-Rabbit IgG (H+L),<br>Alexa Fluor 488 (1:200)	Invitrogen	CAT#A21206<br>RRID:AB_2535792
Donkey anti-Mouse IgG (H+L),<br>Alexa Fluor 488 (1:200)	Invitrogen	CAT#A-21202<br>RRID: AB_141607
Donkey anti-Goat IgG (H+L),<br>Alexa Fluor 488 (1:200)	Invitrogen	Cat#A-11055<br>RRID: AB_2534102
Donkey anti-Rabbit IgG (H+L),<br>Alexa Fluor 546 (1:200)	Invitrogen	CAT#A10040<br>RRID: AB_2534016
Donkey anti-Mouse IgG (H+L),<br>Alexa Fluor 546 (1:200)	Invitrogen	CAT#A10036<br>RRID: AB_2534012

Donkey anti-Goat IgG (H+L),<br>Alexa Fluor 546 (1:200)	Invitrogen	CAT#A-11056<br>RRID: AB_2534103
Donkey anti-Goat IgG (H+L),<br>Alexa Fluor 647 (1:200)	Invitrogen	CAT#A-21447<br>RRID:AB_2535864
Donkey Anti-Goat IgG<br>H&L (HRP) (1:5000)	Abcam	CAT#ab205723<br>RRID:AB_3065024
Goat Anti-Mouse IgG<br>H&L (HRP) (1:5000)	Abcam	CAT#ab6789<br>RRID:AB_955439
Goat anti-Rabbit<br>IgG-HRP antibody (1:5000)	Jackson	CAT#111-035-003<br>RRID: AB_2313567
Bacterial and Virus Strains		
pAAV-GfaABC1D-EGFP-WPRE<br>(AVV2/9，titer：1.68×10^{12} V.G/mL)	OBiO, Shanghai	CAT#H13498
pAAV-GfaABC1D-EGFP-P2A-Cre-WPRE<br>(AVV2/9，titer：1.35×10^{12} V.G/mL)	OBiO, Shanghai	CAT#H7033
rAAV-hSyn-CRE-EGFP-WPRE-hGH polyA<br>(AVV2/9，titer：5.35×10^{12} V.G/mL)	BrainVTA, Wuhan	CAT #PT-1168
rAAV-hSyn-EGFP-WPRE-SV40 polyA<br>(AVV2/9，titer：5.02×10^{12} V.G/mL)	BrainVTA, Wuhan	CAT #PT-0905
AAV-hEF1a-fDIO-mCherry-WPRE-pA<br>(AVV2/9，titer：1.75×10^{12} V.G/mL)	Taitool, Shanghai	CAT#S0553-9
AAV-hEF1a-fDIO-hM4D(Gi)-<br>mCherry-ER2-WPRE-pA<br>(AVV2/9，titer：1.62×10^{12} V.G/mL)	Taitool, Shanghai	CAT#S0336-9-H20
rAAV-EF1α-DIO-*Ptpn6*-P2A-mCherry<br>-WPRE-pA<br>(AVV2/9，titer：1.42×10^{12} V.G/mL)	BrainVTA, Wuhan	Custom-designed
pAAV-GfaABC1D-EGFP-sh*Ptpn6-1*<br>(AVV2/9，titer：1.03×10^{12} V.G/mL)	SunBio, Shanghai	Custom-designed
pAAV-GfaABC1D-EGFP-sh*Ptpn6-2*<br>(AVV2/9，titer：1.65×10^{12} V.G/mL)	SunBio, Shanghai	Custom-designed

pAAV-GfaABC1D-EGFP-sh*Scramble* (AVV2/9，titer：1.49×10^{12} V.G/mL)	SunBio, Shanghai	Custom-designed
pAAV-GfaABC1D-EGFP-P2A-Cre-sh*cxcl1 0* (AVV2/9，titer：4.98×10^{12} V.G/mL)	SunBio, Shanghai	Custom-designed
Chemicals, Peptides, and Recombinant Proteins		
Alexa Fluor™ 647 Phalloidin	Thermo Scientific	CAT#A22287
Clozapine N-oxide	Sigma	CAT# C0832<br>CAS 34233-69-7
Evan's Blue	Sigma	CAT#E2129<br>CAS 314-13-6
Fludarabine	Med Chem Express	CAT#HYb0096
NBI-74330	Med Chem Express	CAT#HY-15320<br>CAS 855527-92-3
NeuroTrace™ 435/455 Blue Fluorescent Nissl Stain (1:200)	Invitrogen	CAT#N21479
PTP inhibitor III	Calbiochem	CAT#540210<br>CAS 29936-81-0
Tamoxifen	Sigma	CAT#T5648
TRITC-Dextran	Thermo Scientific	CAT#T1162
Corn oil (Sterile)	Abcone	CAT#C25834
Kits		
KAPA HotStart Mouse Genotyping Kit	Sigma	CAT#KK7352

Micro BCA™ Protein Assay Kit	Thermo Scientific	CAT#23235
Mouse CXCL10/IP-10/CRG Elisa	R&D systems	CAT#DY466-05
SuperSignal™ West Femto Sensitivity Substrate	Thermo Scientific	CAT#34095
Experimental Models: Organisms/Strains		
Aldh1L1^{CreERT2}	Southern Medical University	Gift from Prof. Tianming Gao's group
Som^{flpo} mice	The Jackson Laboratory	JAX#028579
Ptpn6$^{flox/flox}$	The Jackson Laboratory	JAX# 21160
Software and Algorithms		
Clampfit	Molecular Devices	https://www.moleculardevices.com/
CytExpert	Beckman	https://www.beckmancoulter.com/
Fiji	NIH	https://fiji.sc/
Illustrator CS6	Adobe	https://www.adobe.com/products/photoshop.html
Image Lab	BIO-RAD	https://www.biorad.com/Image-Lab-Software-PC
IMARIS 10.0	Bitplane	https://imaris.oxinst.com/packages
Photoshop CC	Adobe	https://www.adobe.com/products/photoshop.html
Prism 8.0	GraphPad	https://www.graphpad-prism.cn/?c=i&a=prism

R Studio	N/A	https://posit.co/products/open-source/rstudio/
