# Supplementary material for: Loss of SHP1 in Spinal Astrocytes Triggers T‐Lymphocyte Infiltration and Nociceptive Hypersensitivity
Source: Adv Sci (Weinh). 2026 Aug 3:e76932. Online ahead of print. doi: 10.1002/advs.76932 (PMC13430923; doi:10.1002/advs.76932)

# Western blotting original bands for Fig. 2.6.7.and 8; Fi.g S3, and S6

## Figure 2G

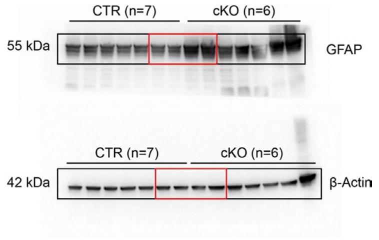


## Figure 6C

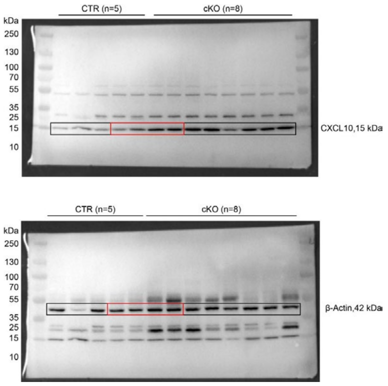


## Figure 7D

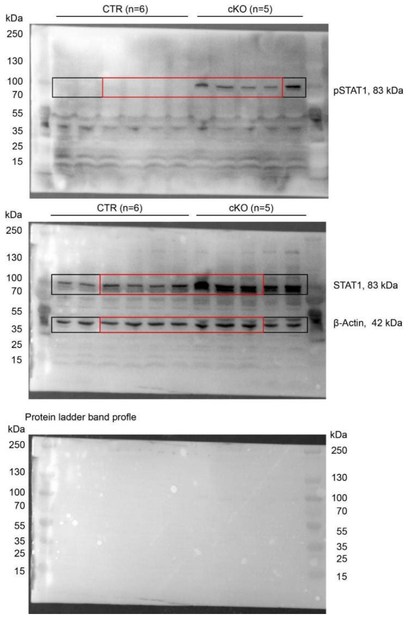


## Figure 7F

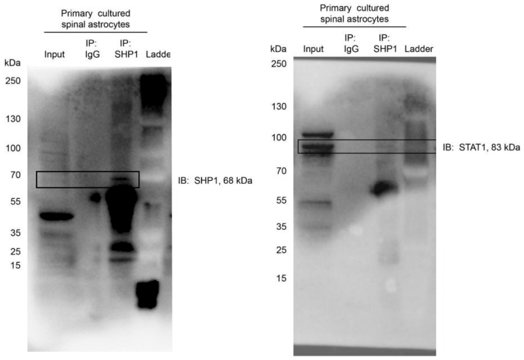


## Figure 7G

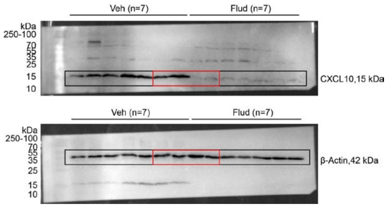


## Figure 8A (1w)

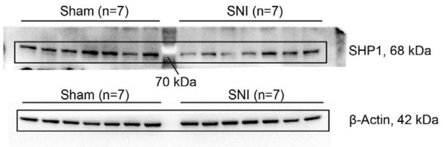


## Figure 8A (2w)

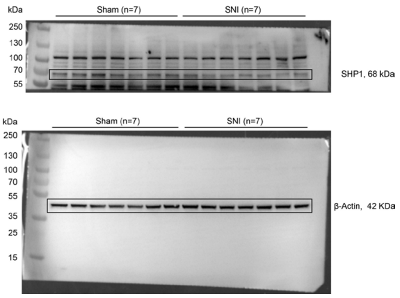


## Figure 8B (1w)

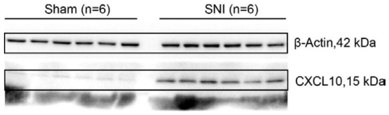


## Figure 8B (2w)

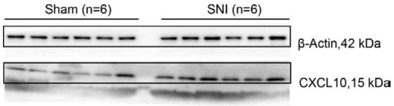


## Figure 8G

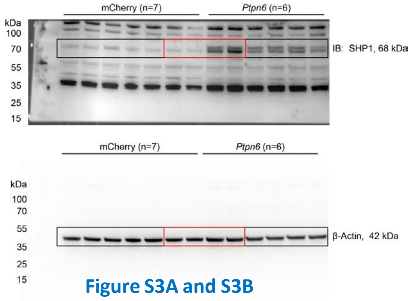


## Figure S3A and S3B

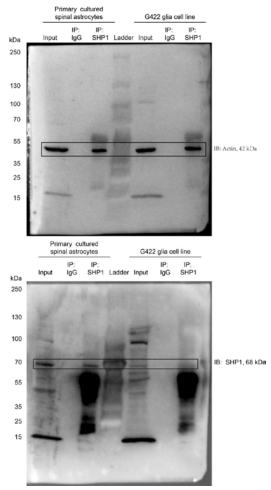


## Figure S3F

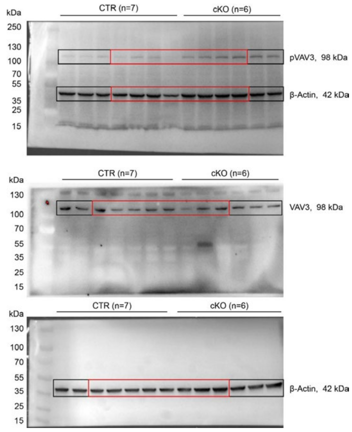


## Figure S3G

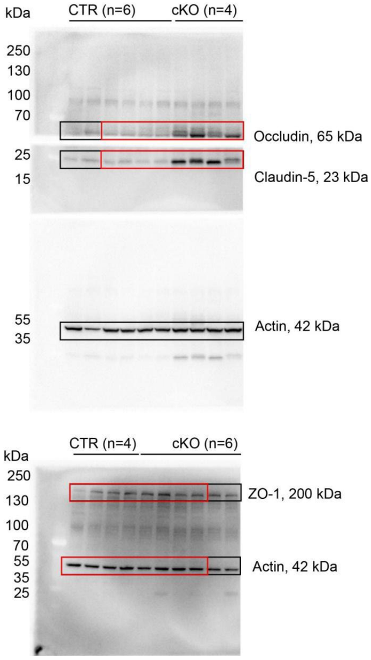


## Figure S6A

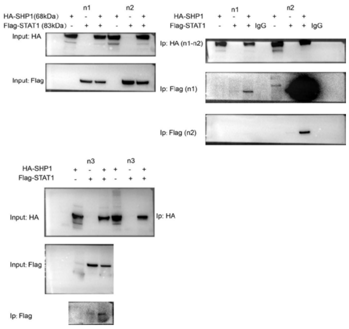


## Figure S6C

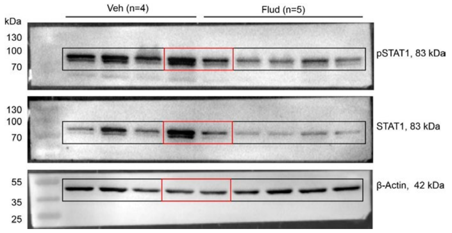

Supplement: Supplementary file 2 — Supporting File 2: advs76932‐sup‐0002‐DataSet.zip. [file ADVS-9999-e76932-s002.zip › Supplemental Figure-Western blotting orginal bands-R3.pdf]
